# UHPLC-OrbiTrap MS Characterization of Phenolic Profiles in French Marigold Extracts and Analysis of Their Antifeedant Activity against Colorado Potato Beetle

**DOI:** 10.3390/plants11030407

**Published:** 2022-02-01

**Authors:** Nina Devrnja, Uroš Gašić, Sanja Šajkunić, Aleksandar Cingel, Sofija Stupar, Ljiljana Tubić, Jelena Savić

**Affiliations:** Institute for Biological Research “Siniša Stanković”—National Institute of Republic of Serbia, University of Belgrade, Bulevar despota Stefana 142, 11060 Belgrade, Serbia; uros.gasic@ibiss.bg.ac.rs (U.G.); sanja.sajkunic@ibiss.bg.ac.rs (S.Š.); cingel@ibiss.bg.ac.rs (A.C.); sofija.stupar@ibiss.bg.ac.rs (S.S.); tubic@ibiss.bg.ac.rs (L.T.)

**Keywords:** biological insecticides, insect protease activity, *Leptinotarsa decemlineata* Say, phytochemical profile, *Tagetes patula* L.

## Abstract

French marigold is an aromatic plant rich in polyphenolic secondary metabolites, which pesticidal potential was examined in this study. Ultra-high-performance liquid chromatography (UHPLC) connected with OrbiTrap mass spectrometer (MS) identified 113 phenolics and revealed the most detailed phytochemistry of French marigold published so far. Depending on plant material (flowers or leaves) and solvents used for extraction (water, methanol, dichloromethane), the phenolic composition varied. Methanol extract of flowers, with 89 identified phenolics and high antioxidant activity statistically comparable with positive control Trolox, was chosen for testing of antifeedant potential against the 3rd and 4th instars of Colorado potato beetle (CPB). A significant reduction in final body mass of 4th larval stage fed with potato leaves coated with methanol extract of flowers in the concentration of 10 mg/mL was observed (157.67 mg vs. 182.26 mg of controls fed with non-treated leaves). This caused delayed molting since treated larvae reached the maximal mass a day after controls and this delay persisted during the entire larval development. Continuous feeding caused a 25% decline in digestive proteolytic activity of the 4th instar in comparison to controls. The results suggest that French marigold methanol extract of flowers could be proposed as a promising antifeedant for CPB management, with an impact on the reduction in the environmental footprint associated with synthetic pesticide application.

## 1. Introduction

The ultimate goal of modern agriculture, dictated by the inexorable growth of the human population, is the increase in the yield and quality of food. According to the estimation of the United Nations (UN) Food and Agriculture Organization (FAO), between 720 and 811 million people faced severe food insecurity in 2020 [1]. Moreover, this report states that around 118 million more people suffered from hunger in 2020 compared to 2019, due to the trembled economic situation caused by the COVID-19 pandemic, and the scenario will be further complicated by the enduring effects of the pandemic. However, intensification of food production is frequently related to dramatic environmental footprints that lead to climate change, water scarcity, land degradation, and deforestation. One of the striking issues that seriously jeopardizes the sustainable approach in agriculture worldwide is the contamination of the environment and produced food with synthetic chemicals used for pest control. Around 0.4 million tons of insecticides produced annually [2] are suspected of causing different adverse effects on the environment [3,4,5] and biodiversity [6]. These harmful chemicals also bring high risks to human health [7], with reports claiming that about 385 million cases of unintentional acute pesticide poisoning occur annually worldwide including around 11,000 fatalities [8]. On the other hand, the most persistent and destructive pests usually possess the ability to overcome the negative effects of the chemicals due to high adaptability, and this has often prompted the increase in quantities of applied pesticides and severe pollution [9].

Searching for sustainable solutions promotes integrated pest management (IPM) that implies the replacement of synthetic chemicals with products from biological sources. Plant-derived active substances, being non-toxic to crop plants and harmless to human and animals’ health, with systematic activity in low concentrations and easy biodegradability with a short half-life, have emerged to be especially favorable for usage in IPM [10,11]. Among a plethora of plant secondary metabolites, phenolic and polyphenolic compounds are responsible for potent antioxidant [12,13] and pesticidal activities [14,15], and are considered to be desired components of botanical insecticides. These compounds cause acute toxicity but also act as antifeedant, growth-inhibiting, and antiovipositional agents [16,17,18,19] which influence the growth and development of pests, reducing their harmfulness. In addition, phenolics can turn their antioxidant into prooxidant activity which leads to lethal oxidative damage to pests’ cells.

French marigold (*Tagetes patula* L.), an aromatic plant grown worldwide for ornamental purposes, is one of the plant species whose secondary metabolites exhibit potent pesticidal activities [20,21,22,23,24]. Plants are especially rich in flavonoids, a class of polyphenolic secondary metabolites, with the highest diversity and quantities reported in flowers [25]. The most abundant flavonoids in French marigolds are patuletin, quercetin, quercetagetin, patulitrin, luteolin, allopatuletin, and kaempferol [26,27,28,29,30,31], present either as aglycones or as glycosylated forms with different sugar moieties. Extracts obtained from flowers or leaves of French marigold have already been tested against pests from Hemiptera–western tarnished plant bug *Lygus hesperus* Knight and the whitefly *Bemisia tabaci* Gennadius [24], as well as from Diptera–mosquitoes *Aedes aegypti* L. and *Anopheles stephensi* Liston [32], and cabbage maggot *Delia radicum* L. [33]. In most cases, extracts incorporated into an artificial diet significantly reduced the survival of feeding larvae and this effect was observed in a dose-dependent manner. However, the data regarding the chemical composition of used French marigold extracts are usually limited or completely lacking, aggravating the elucidation of the potential mode of action of extracted phytochemicals, as well as the realization of their industrial utilization as botanical insecticides.

Colorado potato beetle (CPB, *Leptinotarsa decemlineata* Say) is the principal insect pest of potato (*Solanum tuberosum* L.) and many other cultivated species, such as tomato (*S. lycopersicum* L.), eggplant (*S. melongena* L.), cabbage (*Brassica oleracea* L.), pepper (*Piper nigrum* L.), tobacco (*Nicotiana tabacum* L.), but also wild species, of which many serve as a reservoir for infestation [34]. Spread throughout the Northern hemisphere, voracious larvae and adults could significantly defoliate potato plants before potato tuber initiation, leading to 40–80% yield losses [35]. CPB is one of the pests with the highest adaptability, which possesses the extraordinary ability to develop resistance to insecticides [9]. Currently, this pest is resistant to about 56 commercially available products belonging to organophosphates, carbamates, chlorinated hydrocarbons, pyrethroid, and neonicotinoid insecticides [36]. For over 70 years, valuably forces have been invested in the fight against this “super pest” and these efforts are presumably responsible for creating the modern insecticide industry [37]. However, only a tenth of commercially available botanical products are currently present on the market for use against CPB, e.g., Hot Pepper Wax™ from *Capsicum annuum* L., BioNeem^®^, NEEMIX^®^, Margosan-O and Azatin XL Plus from *Azadirachta indica* A. Juss., Pyola™ from *Tanacetum cinerariifolium* Sch.Bip. Most of them contain volatile terpenoid compounds and display repellent activity [38], but also act as insect antifeedants, sterilants, and growth disruptors [39]. Few botanical products contain flavonoid compounds, such as isoflavone rotenone isolated from the roots of plants from the Fabaceae family. Rotenone blocks electron transfer in mitochondria to ubiquinone, causing a reduction in cellular oxygen, and the initiation of reactive oxygen species that can damage DNA and other components of the mitochondria [40]. However, rotenone is mildly toxic to humans and other mammals, and the link between rotenone use and Parkinson’s disease has been shown in farmworkers [41], causing the withdrawal of this insecticide from the market. Thus, the search for new sources of botanical insecticides must be intensified.

Plenty of plant extracts have been so far tested against CPB larvae. In the study by Gökçe et al. [42], 30 different plant extracts were tested against 3rd instars. The plant extracts exhibited varying toxicity to the larvae ranging from 0–91% after 24 and 48 h incubation, with *Artemisia vulgaris* L., *Hedera helix* L., *Humulus lupulus* L., *Lolium temulentum* L., *Rubia tinctoria* L., *Salvia officinalis* L., *Sambucus nigra* L., *Urtica dioica* L., *Verbascum songaricum* Schrenk., and *Xanthium strumarium* L. extracts, that resulted in significantly higher mortality than in the control. However, conclusions about the potential mode of action could not be made since the phytochemical composition of tested extracts has not been examined.

Since extracts of French marigold (*Tagetes patula* L.) have not been tested against CPB so far, this research aimed to examine the potential of this plant species as a promising high-yielding source for industrial exploitation and production of bioinsecticide formulation against the CPB. The overarching research goal of the presented study was to obtain rich French marigold extracts from flowers and leaves in solvents with different polarities (water, methanol, dichloromethane) and to compare their phytochemical compositions and in vitro antioxidant potential. In addition, we performed digestive toxicity assays with CPB larvae to examine larvicidal potential and influence on larval development and the digestive activity of the extract with the highest abundance of phenolics.

## 2. Results

### 2.1. Phytochemical Profile of French Marigold Extracts

Extraction of French marigold phenolics from flowers and leaves by three different solvents resulted in extracts with various compositions and an abundance of specific compounds. Phytochemical characterization by ultra-high-performance liquid chromatography connected with OrbiTrap mass spectrometer (UHPLC-OrbiTrap MS) culminated with the identification of 113 compounds in total, present in 6 different extracts, i.e., extracts from both flowers and leaves, each in water, methanol (MeOH), and dichloromethane (DCM) (Supplementary Table S1). A total of 26 phenolic acids and 20 phenolic acid glycosides were detected and identified from the chromatogram (Supplementary Figure S1). The least diverse group was flavonoids (12); however, their glycosides (55) were present in the highest diversity.

**Figure 1:**
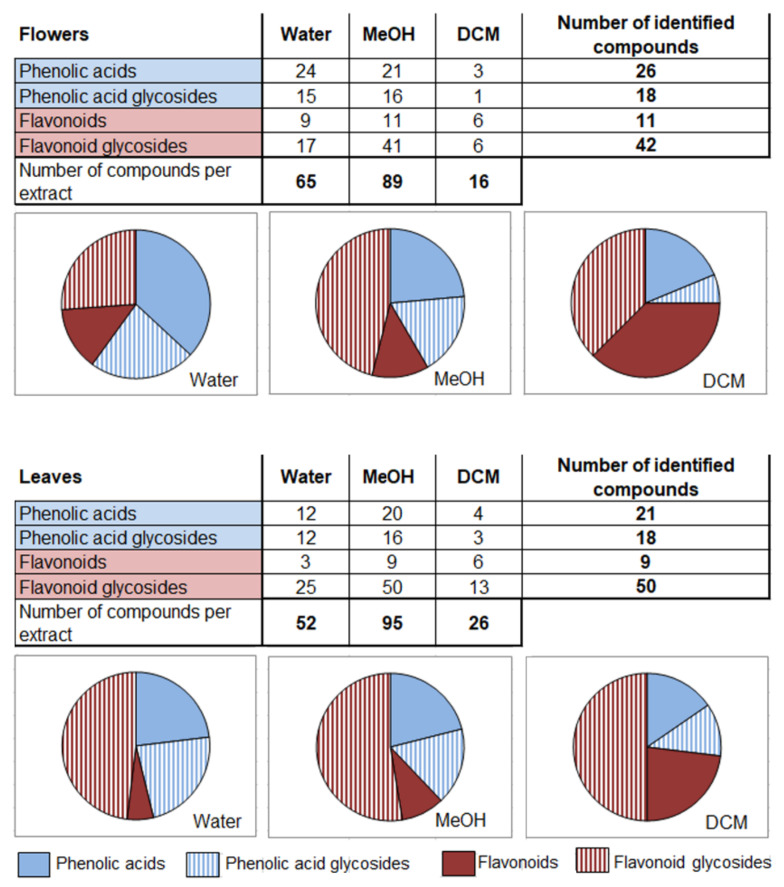
Number of identified phenolic acids, phenolic acid glycosides, flavonoids, and flavonoid glycosides in water, methanol (MeOH), and dichloromethane (DCM) extracts of flowers and leaves of French marigold. Pies represent relative distribution of different groups of phenolics in each extract (number of compounds per extract = 100%).

**Figure 2:**
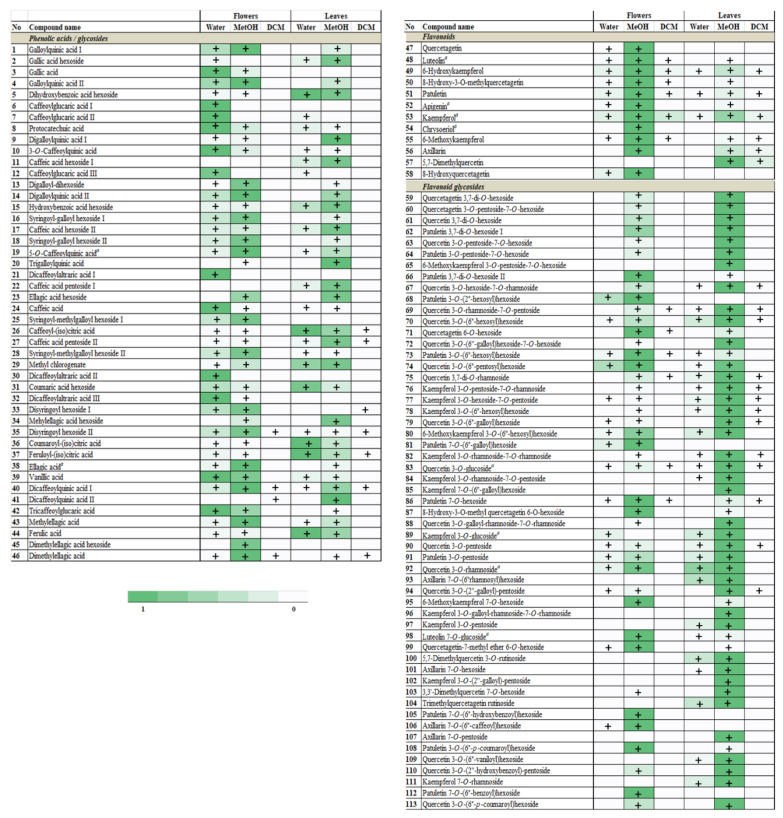
Distribution of different phenolics in extracts of French marigold flowers and leaves obtained in water, methanol (MeOH), and dichloromethane (DCM). Heatmaps represent values for scaled areas obtained from full scan MS for each compound (1 in the color legend corresponds to the maximal peak area detected for each compound).

Solvents differing in polarity extracted diverse compounds belonging to different chemical groups. A similar number of compounds were extracted from flowers and leaves, but the distribution among groups was slightly different (Figure 1). The highest number of different compounds was extracted with MeOH for both flowers and leaves (89 and 95, respectively), while DCM extracted only 16 compounds from flowers and 26 compounds from leaves. Only six compounds, i.e., one phenolic acid (dicaffeoylquinic acid isomer I), one phenolic acid glycoside (disyringoyl hexoside isomer II), three flavonoid aglycones (6-hydroxykaempferol, patuletin, and kaempferol), and one flavonoid glycoside (quercetin 3-*O*-glucoside), were present in all extracts (Figure 2). Some of the flavonoids typical for Tagetes species, such as quercetagetin and 8-hydroxyquercetagetin were identified only in flowers.

The most diverse extracts were obtained by MeOH extraction, with a total of 95 identified compounds in leaves and 89 in flowers (Figure 1).

According to the relative comparison of peak areas from the full scan spectra for each specific compound (Supplementary Table S2), flavonoid aglycones in flowers and flavonoid glycosides in leaves were dominant phenolic groups (Figure 2). On the other hand, water as solvent extracted the highest portion of phenolic acids compared to MeOH and DCM. Dichloromethane extracted the smallest number of compounds (26 and 16, in leaves and flowers, respectively) with flavonoid glycosides as the most diversified group (Figure 1 and Figure 2).

All identified flavonoid glycosides were derivatives of patuletin, quercetagetin, quercetin, luteolin, axillarin, and kaempferol. Insight into MS fragmentation of flavonoid glycosides showed that almost all compounds build glycosylated derivatives at the 3-*O* and/or 7-*O* position, except tagenol B (compound **87**, 8-hydroxy-3-*O*-methyl quercetagetin 6-*O*-hexoside), an uncommon compound with glycoside at the 6-*O* position identified in the MeOH extract of French marigold leaves and flowers (Supplementary Figure S2). Determination of the nature of the interglycosidic linkage between sugars (1 → 2 or 1 → 6 linkage) linked to the flavonoid core was evaluated by studying specific MS^2^ fragments resulting from the gradual cleavage of primary sugar. These mass spectrometry rules in the study of flavonoid glycosides are well known and available in the literature [43,44,45]. Moreover, there are certain MS^2^ fragments that can greatly alleviate doubts about the nature of glycosylation position and for this purpose, the relative intensities of the aglycone part of the molecule ([M-H-sugar]^−^ and its radical ion are observed [43,46,47].

### 2.2. Antioxidant Activity of French Marigold Extracts

Results of total phenolic content (TPC) and free radical scavenging assays of tested extracts in comparison to Trolox as standard are shown in Table 1.

The highest content of total phenolics was measured in the extract obtained by extraction of French marigold flowers in MeOH (81.56 mgGAE/g). TPC of all other extracts was for about 40% (53 mgGAE/g for leaves in MeOH), 50% (37.34, 37.31, and 39.88 mgGAE/g for flowers in DCM, flowers in water, and leaves in water, respectively), or even 60% (29.84 mgGAE/g for leaves in DCM) lower.

As shown in Table 1, the MeOH extract of French marigold flowers demonstrated potent antioxidant activity with IC_50_ values for all three performed assays (DPPH IC_50_ = 0.690 mg/mL, ABTS IC_50_ = 0.489 mg/mL and FRAP IC_50_ = 0.044 mg/mL) being comparable to Trolox (DPPH IC_50_ = 0.013 mg/mL, ABTS IC_50_ = 0.103 mg/mL and FRAP IC_50_ = 0.010 mg/mL). Methanol extract of leaves also exhibited strong DPPH radical scavenging (IC_50_ = 0.152 mg/mL) and FRAP reducing potential (IC_50_ = 0.096 mg/mL), while the activity toward the ABTS radical was significantly lower (IC_50_ = 1.436 mg/mL).

The dichloromethane extract of leaves showed the lowest antioxidant potential according to all performed assays. The IC_50_ values for all tests were the highest measured (DPPH IC_50_ = 5.465 mg/mL; ABTS IC_50_ = 2.578 mg/mL; FRAP IC_50_ = 0.299 mg/mL).

According to Pearson’s coefficient, the negative correlation between TPC and IC_50_ values, reflecting antioxidant potential, was observed for all performed antioxidative assays. The strong correlations were recorded for FRAP (R^2^ = −0.820) and ABTS (R^2^ = −0.799), indicating a significant impact of phenolic compounds in respective antioxidative performances of French marigold extracts. The influence of phenolic compounds in DPPH radical scavenging potential was also present, but that influence was moderate (R^2^ = −0.551).

### 2.3. Effect of French Marigold Extract on Colorado Potato Beetle Larvae

#### 2.3.1. Growth and Development of CPB Larvae

**Figure 3:**
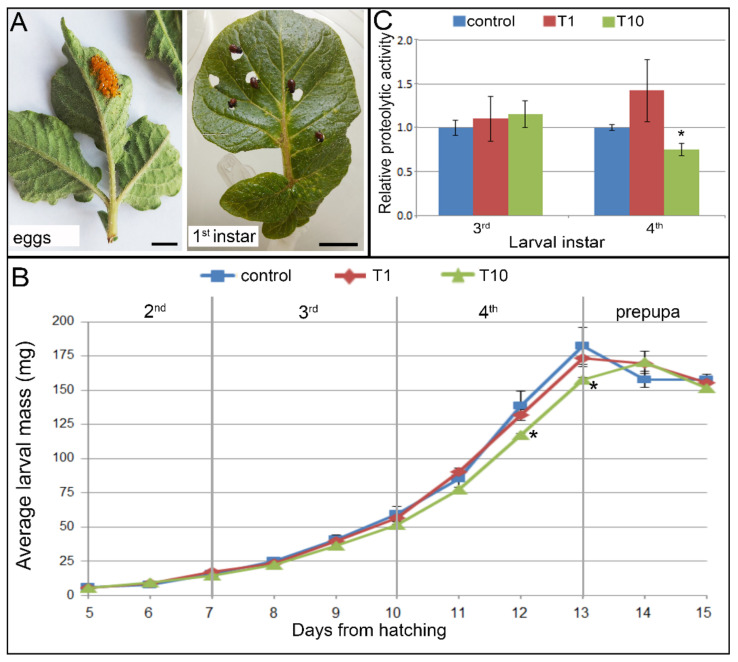
Effect of French marigold methanol extract of flowers on CPB larval growth and digestive activity. Larvae of 2nd instar hatched from eggs (**A**) were fed with potato leaves coated with the extract in concentrations 1 mg/mL (T1) or 10 mg/mL (T10) or with control leaves. Larval mass gain (**B**) was recorded daily during the entire larval development, from the 2nd through 4th instar to the prepupal stage (given stage borders are for controls). Digestive protease activity (**C**) in midguts of 3rd and 4th instars fed with T1 and T10 coated potato leaves was analyzed and presented relative to activity measured in control larvae fed with non-coated leaves. All results are presented as means ± standard errors (*n* = 15 for (**B**) and *n* = 3 for (**C**)), and values with an asterisk (*) were different than corresponding controls according to Student’s *t*-test (*p* ≤ 0.1) for each day of measurement (for (**B**)) or larval stage (for (**C**)) separately. Bars represent 1 cm.

**Figure 4:**
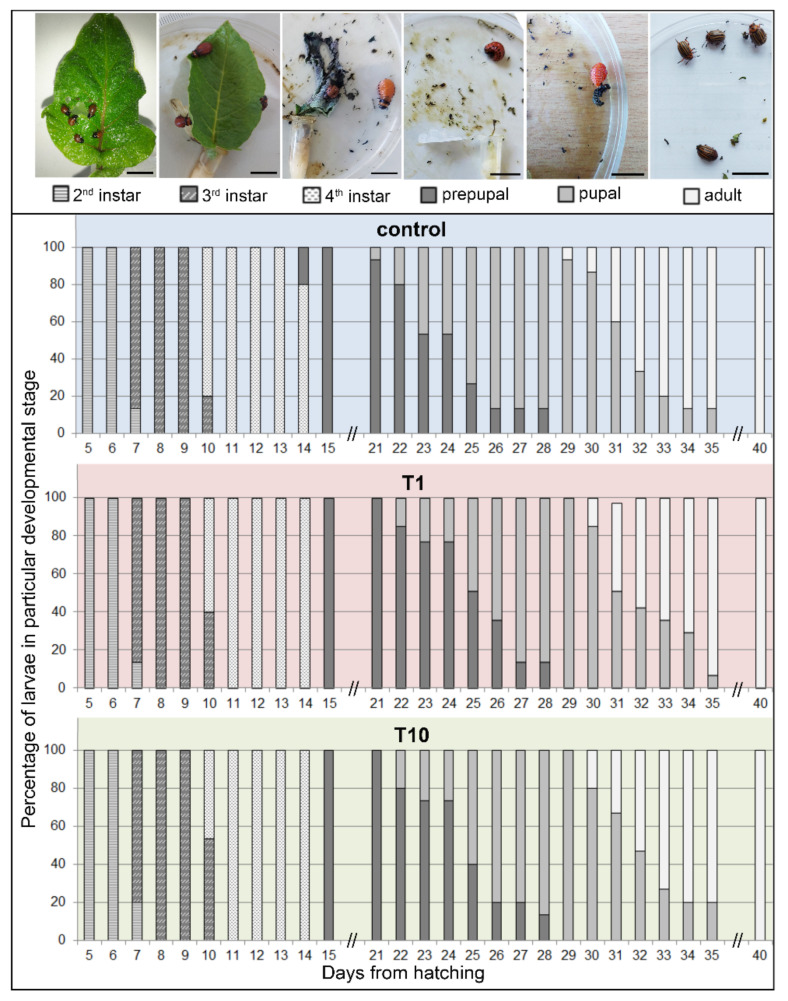
Effect of French marigold methanol extract of flowers on CPB larval development. The potato leaves coated with extract in concentrations of 1 mg/mL (T1) and 10 mg/mL (T10), were used as food for CPB larvae during development from 2nd, through 3rd, and 4th instars. The overall developmental pattern was compared with larvae fed on control leaves. Results are presented as a percentage of larvae in the particular developmental stage for each day during the entire development. Bars represent 1 cm.

Feeding on potato leaves coated with the MeOH extract of French marigold flowers (in concentrations of 1 mg/mL–T1 and 10 mg/mL–T10) altered larval growth and development of CPB larvae when applied at a concentration of 10 mg/mL (Figure 3 and Figure 4). However, the extract did not cause mortality, with survival rates of 100% obtained for all experimental groups. In addition, no significant effect of extract ingestion on pupation and adult emergence has been observed (Figure 4).

Larvae that emerged from egg clusters (Figure 3A) and that entered into the 2nd instar after 4 days were randomly placed on control or T10-coated leaves and mass gain and larval development scored daily. During the 2nd and 3rd instars, all larvae from extract-coated leaves grew and molted synchronized with larvae raised on control leaves (Figure 3 and Figure 4). At the beginning of the 3rd instar, at day 7 from hatching, and day 3 from the start of feeding, all larvae had similar masses (Figure 3B; controls: 15.61 ± 0.88 mg; T1: 17.31 ± 1.14 mg; T10: 14.26 ± 0.55 mg). After 3 days of continuous feeding, at the end of the 3rd instar, T10 larvae were slightly lighter (51.39 ± 0.36 mg) than larvae from controls (59.55 ± 5.26 mg) or T1 (56.31 ± 2.60). This pattern continued, and at the end of the 4th developmental stage, these differences were even more pronounced and became statistically significant according to Student’s *t*-test for T10 at day 12 (117.16 ± 1.18 mg vs. controls 138.59 ± 10.91 mg with *p* = 0.061, α = 0.1, T = 1.95, df = 4) and day 13 (157.67 ± 1.48 mg vs. controls: 182.26 ± 13.52 mg with *p* = 0.072, α = 0.1, T = 1.81, df = 4). For larvae fed on control and T1 leaves, these were the maximal reached masses, and after that, larvae decreased feeding, and body masses reduced at the onset of pupation. On the other hand, larvae fed with leaves coated with a French marigold extract at a concentration of 10 mg/mL, continued with feeding, reaching the maximal mass a day after.

This slower mass gaining also affected the dynamic of larval development (Figure 4). All larvae exhibited synchronized molting during the 2nd and the first 2 days of the 3rd instar, regardless of the type of leaves consumed. However, the type of consumed leaves influenced the number of larvae that molted to the 4th instar. On day 10 from hatching, 80% larvae fed with control leaves, 60% of larvae fed on leaves coated with a lower concentration of extract (T1), and only 46.67% of once fed on T10 leaves, passed into the 4th instar. Thus, the duration of the 4th instar differed related to consumed leaves. These differences conditioned the appearance of the first pupae and adults earlier in control than in treated groups. However, after 40 days all analyzed larvae finished development as adults.

#### 2.3.2. Digestive Proteolysis in Larvae Fed with French Marigold Extract-Coated Potato Leaves

Analysis of digestive proteases of CPB larvae showed that feeding on potato leaves coated with the MeOH extract of French marigold flowers affected nutritional physiology (Figure 3C). The effect of prolonged feeding was analyzed in the 3rd and 4th instars, observing the larvae that had been fed continuously from the 2nd instar. After 6 days of continuous feeding, the digestive proteolytic activity of 3rd instars’ midgut slightly increased compared to controls in both T1 and T10 larvae (with 10 and 15% increment, respectively).

The trend of exalted proteolytic activity was also noticed in larvae of the 4th instar fed on leaves coated with the lower concentration of extract (T1). The proteases activity was about 50% higher than in controls. However, chronic continuous feeding with leaves coated with more concentrated extract (T10) caused a 25% digestion decline (*p* = 0.078, α = 0.1, df = 4.13).

## 3. Discussion

The selection of plant species with potential insecticidal properties is usually based on traditional habits, growers’ experience, and recommendations, or scientific literature data. French marigolds are traditionally claimed as companion plants having an impact on densities of insect pest populations in potato fields, mainly due to repellent activity [39]. Some authors also reported the antifeedant potential of essential oils or extracts, made from herbs or flowers, on insect pests [23,24,31]. However, the lack of information on phytochemical compositions of these botanical insecticides could be one of the major discouraging steps in a long “journey” from laboratory research to industrial production, market outbreak, and commercial application.

Phytochemical analyses of six French marigold extracts, made from either flowers or leaves extracted each in water, MeOH, or DCM, revealed that the solvents used strongly altered the presence of compounds from different groups of phenolics, i.e.,, phenolic acids and flavonoids, in glycosylated forms or not. These results are in line with theoretical expectations since the literature data claim that the phytochemical composition and subsequent biological potential of plant extracts strongly depend on solvents used for extraction, regardless of the method (i.e.,, maceration, microwave-assisted extraction, ultrasound-assisted extraction, accelerated solvent extraction) employed [48,49]. The choice of solvent depends mainly on the nature of the desired bioactive compounds [50].

Employment of comprehensive UHPLC-OrbiTrap MS characterization enabled the detection of a significant number of phenolic compounds in French marigold extracts. Identification of 113 phenolics in obtained extracts revealed the most detailed phytochemistry of French marigold published so far as to our knowledge. Although genus *Tagetes* is known for species with proven industrial potential, the majority of previous studies focusing on chemical characterization of different extracts commonly revealed a smaller number of compounds with an incomplete picture of phytochemical diversity responsible for demonstrated biological activities. In the work of Bhave et al. [51], French marigold flowers extracted in acidified MeOH, and characterized by HPLC-MS/MSC, revealed a total of 12 flavonoids identified from 26 peak compounds. Politi et al. [52] used the same method and identified kaempferol and patuletin, along with 10 glycosylated flavonoids in the ethanolic extract of aerial parts.

Among the varied flavonoids identified in this study, patuletin, kaempferol, and 6-hydroxykaempferol were detected in all extracts irrespective of the solvent used. Interestingly, quercetin, as one of the dominant flavonoids in plants, was not detected in French marigold extracts as aglycon, while the wide spectrum of its 3-*O* or 7-*O*-glycosides was present in all leaf extracts. Insight into MS fragmentation of flavonoid glycosides showed that almost all compounds build glycosylated derivatives at the 3-*O* or 7-*O* position, which can be deduced from the intensities of the basic MS^2^ peaks [53]. However, a study of the literature has shown that certain glycosides which can be found in some plants from Tagetes genus form a glycoside derivative at the 6-*O* position [54]. Thus, for example, the presence of 8-hydroxy-3-*O*-methyl quercetagetin 6-*O*-hexoside (compound **87**), which is trivially called tagenol B [55], in the MeOH extracts of French marigold leaves and flowers was confirmed in this paper.

Determination of total phenolic content (TPC) using standard Folin–Ciocalteu quantification correlated with the results of the UHPLC analysis and proved the most abundant phenolics in methanol extracts of both leaves and flowers. In addition, these extracts demonstrated the highest antioxidant potential in ABTS, DPPH, and FRAP assays, which was expected since typical phenolics that possess antioxidant activity are phenolic acids and flavonoids. Interestingly, the water extract of flowers exhibited strong antioxidative activity against DPPH radicals, probably due to the abundance of phenolic acids. According to structure–activity relationship (SAR) analyses, often used to find correlations between biological activities and physicochemical properties of compounds, the antioxidant potential of flavonoids depends strongly on the number and position of hydroxyl groups in the molecule [56,57]. Thus, phenolic hydroxyls seem to be the core of the antioxidant ability of phenolics. Along with hydroxyls, the presence of a methoxy group (–OCH_3_), the dihydroxylated B-ring (catechol structure), unsaturation, and 4-oxo function in the C-ring also increases the antioxidant capacity and widens the biological activities of the compounds [58,59]. This is exactly the structure of French marigold-dominant flavonoid patuletin, which gives it strong antioxidant potential, and considered to be responsible for the proven anti-inflammatory and cytotoxic [60,61], antimicrobial [62], analgesic [29], antirheumatoid arthritis [63] activities. Expectedly, patuletin was not the only flavonoid responsible for the potent antioxidant activity of French marigold extracts. The presence of compounds with similar structural characteristics significantly contributed to and increased the antioxidant potential, especially in the MeOH extract. Quercetin that was present in numerous glycoside forms, especially in leaves, possesses the aforementioned structure, with the only difference regarding patuletin being on the C6 position, where it has a methoxy group. The third compound with C6–OH, quercetagetin, was present only in MeOH extracts of flowers, which exhibited the strongest antioxidant potential in all three tests performed. The reactive –OH group of this compound unequivocally contributes to the antioxidant activity of this extract, which is in line with reported claims that at least two hydroxyl groups in ring B enhance the antioxidant capacity of hydroxylated flavonoids [64]. The proposed mechanism relies on the ability of hydrogens and electrons donated by ring B hydroxyl groups to react with hydroxyl, peroxyl, and peroxynitrite radicals, resulting in the formation of relatively stable flavonoid radicals.

Although phenolics exhibit a strong antioxidative potential, on some specific occasions they can turn their action into prooxidants, especially in a system containing redox-active metals. For example, the presence of iron or copper catalyzes their redox cycling and may lead to the formation of phenolic radicals, which could cause oxidative damage to the cells. Barbehenn et al. [65,66] reported that in the lumen of the insects’ gut, phenolics frequently show a prooxidant activity, as they generate substantial levels of superoxide ion (O_2_^−^), hydrogen peroxide (H_2_O_2_), and organic hydroperoxides (ROOH). Direct quantification of phenolic oxidation showed that phenolic compounds from the leaves of red oak and sugar maple were oxidized in the midgut fluids of *Malacosoma disstria* Hübner and *Orgyia leucostigma* J. E. Smith caterpillars. In addition, the concentration of the antioxidants in matrix environments could also favor the prooxidant properties of phenolics [67]. Oxidized flavonoids, such as quercetin and kaempferol, have been shown to induce DNA damage and lipid peroxidation in the presence of a transition metal [68,69].

As larvae of CPB are extremely voracious foliage feeders, 3rd and 4th instars ingesting from 15 to 77% of the total leaves consumed during the entire development [70] feeding on leaves coated with extracts rich in phenolics could impair their nutritional physiology and overall development. However, a bioassay performed on CPB 3rd instars fed with potato leaves coated with pure quercetin did not demonstrate any effect of this compound on feeding deterrence, larval growth, food consumption, or conversion of digested food into insect body mass [17]. Nonetheless, in a subsequent study by Pavela [71], crude MeOH extracts obtained from 42 plant species (of 75 tested) showed effective feeding deterrence in the range of 50–95% against 4th CPB instars. All extracts exhibited antifeedant potential through inhibition of feeding that did not kill the pest but rather limited its development potential considerably. This is considered a favorable characteristic since these compounds and/or crude extracts do not cause strong selection pressure on pest populations that could lead to the survival and spread of resistant individuals, and have selective actions against beneficial organisms, such as pest parasites and predators, as well as pollinators [16].

The basic concept of antifeedants usage in the battle against CPB is to spray them on potato plants to deter feeding and reduce the damage of potato foliage. One of the most successful is neem extract, a well-recognized botanical insecticide, which exhibits the potent antifeeding activity against CPB, with the magnitude of the effects depending on the dominant life stage present during the application, as well as on the attack intensity [72,73]. It was shown that some phenolics from plant extracts act as inhibitors of glucose transport and decrease the glucose level [74,75], which can also alter normal development since it is known that it serves as an energy source to the larval and pupal stages. Thus, the antifeedant activity of the MeOH extract of French marigold flowers in this experiment was tested on detached potato leaves coated with it. The observed reduction in final body mass of 3rd and 4th instars fed with concentrated extract (10 mg/mL) resulted in delayed molting. Slower attainment of critical body mass resulted in insufficient stimulation of stretch receptors in the insect abdomen and poor secretion of prothoracicotropic hormone (PTTH), which is necessary for a larva to molt [76]. A study by Devi and Bora [77] showed that the effect on growth and development was accompanied by changes in the macromolecular (carbohydrates, lipids, and proteins) levels in the mosquitoes treated with phenolic extract of *Ziziphus jujuba* Mill. plants. These authors conclude that the appropriate accumulation of these nutrients during larval development regulates juvenile hormone synthesis responsible for activation of maturation. It is also possible that luteolin contributed to the effect of delayed molting since it was shown that this compound can inhibit enzymatic activity and prevent the growth of different insect species’ larvae [78].

Results of relative proteolytic activity showed some alternation in treated larvae compared to controls but without significance. These alternations may be the result of physicochemical changes caused by phenolic compounds that may determine biological properties, including the digestibility and utilization of food proteins as well as the activity of digestive enzymes [79,80].

The observed developmental pattern could also be related to the flavonoids, but the presence of phenolic acids, for example, ellagic and caffeoylquinic, certainly contributed to this activity. Thus, due to the presence of quercetagetin, the MeOH flower extract showed the highest antioxidant potential. This extract was selected for antifeedant bioassay on CPB. However, the precise mode of antifeedant action is not fully understood for both complex extract mixtures or pure compounds and must be a primary goal of future research among basic and applied entomologists interested in insect–plant interactions or the control of herbivore pests.

## 4. Materials and Methods

### 4.1. Plant Material and Extracts Preparation

French marigold (*Tagetes patula* L.) plants (seeds purchased from Semenarna Ljubljana, Slovenia) were collected at the blooming stage from the private garden in Belgrade nearby (village Jajinci, Serbia). Plant material was left in shade at room temperature. After 2 weeks, dried material was separated into flowers and leaves portions, and ground to a fine powder in a mechanical blender. Maceration was conducted in different polar solvents, i.e.,, water, methanol (MeOH), and dichloromethane (DCM), all at a 1:10 ratio (w/v). All solvents were added directly to the plant material and left on a magnetic stirrer for 2 h. Water macerate was heated at 60 °C during that period to provide better extraction. After that, maceration was continued in the dark for the next 24 h. The extraction was finished by 3 × 5 min of ultra-sonication (Sonorex, Bandelin, Berlin), and obtained extracts were subsequently filtrated through Whatman No1 filter paper. MeOH and DCM were removed by rotor-evaporator (Buchi R-210, Flawil, Switzerland), while aqueous extract was freeze-dried by lyophilization (LH Leybold, Lyovac GT2, Frenkendorf). A total of six dry crude extracts, obtained from flowers and leaves, both in 3 different solvents, were stored at 4 °C in sterile glass bottles protected from light, until further use.

### 4.2. UHPLC-OrbiTrap MS Assessment of French Marigold Extracts

For chromatographic analysis, all six dry crude extracts were dissolved in MeOH at a concentration of 1 mg/mL. Analysis was carried out on the Accela ultra-high-performance liquid chromatography (UHPLC) system, connected with linear trap quadrupole (LTQ)-OrbiTrap mass spectrometer. Heated electrospray ionization (HESI) was applied (Thermo Fisher Scientific, Bremen, Germany). Separation was performed by the Syncronis C18 column (100 × 2.1 mm, 1.7 μm particle size). The chromatographic and mass spectrometry (MS) settings were the same as previously described in the literature [81].

Ten polyphenols were identified using analytical standards, while the identification of other components (in the absence of standards) was carried out according to their monoisotopic mass (obtained by full scan (FS) analysis) and MS^n^ fragmentation, and confirmed by the literature data of metabolites identified in various Tagetes species [51,55,82,83,84]. RStudio software (version 1.3.1093) was used for the MS data evaluation.

The molecular formulas of compounds were obtained from exact masses of peaks from FS analysis, while the tentative structures of compounds were proposed by examination of its MS^2^, MS^3^, and MS^4^ fragmentation.

### 4.3. Determination of Total Phenolic Content (TPC)

The total phenolic content (TPC) of French marigold extracts was assessed based on the modified method of Singleton and Rossi [85]. Dry crude extracts were first dissolved in MeOH in the concentration of 5 mg/mL. Folin–Ciocalteu (FC) reagent was prepared as 10% (*v*/*v*) aqueous solution and mixed with extracts in a 5:1 ratio (*v*/*v*; 500 µL of FC and 100 µL of extract of the certain concentration), followed by the addition of 400 µL of 7.5% (*v*/*v*) sodium carbonate after 4 min. The absorbance at 765 nm was read after 2 h of incubation in the dark. TPC was calculated from the standard curve obtained for gallic acid (GA) and presented as milligrams of GA equivalent (GAE) per gram of dry extract (DW).

### 4.4. Estimation of In Vitro Antioxidant Potential of French Marigold Extracts

#### 4.4.1. DPPH Assay

Antioxidant activity of French marigold extracts was determined by the method of Brand-Williams et al. [86] with slight modifications using 1,1-diphenyl-2-picrylhydrazyl (DPPH), a stable free radical which can be neutralized by antioxidants from extracts. Briefly, methanol-dissolved crude extracts in different concentrations were mixed with 50 µM methanolic solution of DPPH in a 1:1 ratio (*w*/*w*) and incubated in the dark for 30 min, before measuring the absorbance at 517 nm. Trolox ((+)-6-hydroxy-2,5,6,7-tetramethylchromane-2-carboxylic acid) was used as a positive control. The percentage of DPPH radical inhibition was calculated with the following equation:

% inhibition = [(*A*_0_ − *A*_1_)/*A*_0_] × 100 (1)

where *A*_0_ is the absorbance of DPPH methanol solution and *A*_1_ is the absorbance of this solution with extract added.

For every extract and Trolox, a standard curve was formed from values obtained for the inhibition percentage of every concentration. Based on linear regression of plots, IC_50_ values (which represent a concentration of extract required for 50% inhibition of free radicals) were calculated.

#### 4.4.2. ABTS Assay

ABTS (2,2′-azino-bis(3-ethylbenzothiazoline-6-sulfonic acid)) radical caption assay [87] was used for the determination of the antioxidant potential of French marigold extracts. The working concentration (2.45 mM) of ABTS^+^ free radical was formed by oxidizing 7 mM aqueous solution of ABTS with 4.9 mM solution of potassium persulfate in ratio 2:1. The mixture was then kept in the dark at room temperature for 16 h to allow free radical generation and was then diluted with 80% (v/v) methanol (1:44, v/v). The reaction mixture was formed by mixing 950 µL of ABTS reagent and 50 µL of plant extract in a certain concentration. After 30 min of incubation in the dark at room temperature, the characteristic dark green color of ABTS radical vanished to light green in the presence of antioxidants from the extract, and the decrease in absorbance at 734 nm was measured (Multiskan FC microplate photometer, Thermo Fisher Scientific, Vantaa, Finland). Trolox was used as a positive control. The results were expressed as IC_50_ values which represent the concentration of extract that inhibits 50% of ABTS^+^ radical.

#### 4.4.3. FRAP Assay

In vitro assessment of antioxidant power of French marigold extracts was carried out by the Ferric reducing antioxidant power (FRAP) assay [88], based on the reduction of ferric to ferrous ion at low pH. FRAP reagent was formed by mixing 100 mL of 300 mM acetate buffer at pH 3.6, 10 mL of 10 mM ferrous-tripyridyltriazine (TPTZ) solution, and 10 mL of 20 mM FeCl_3_ × 6H_2_O solution. In 950 µL of FRAP reagent, 50 µL of plant extract in various concentrations (0.01–1 mg/mL) was added and the mixture was incubated in the dark for 30 min at room temperature. Trolox was used as a positive control. The increase in absorbance of formed colored TPTZ complex was measured at 594 nm on an ELISA microplate reader. The results were expressed as IC_50_ values.

### 4.5. Antifeedant Bioassay with Colorado Potato Beetle

#### 4.5.1. Insect Rearing and Feeding with Potato Leaves Coated with French Marigold Extract

Leaves with CPB egg clusters were collected from field-grown, pesticide-untreated potato plants (cultivar Désirée) during summer 2019 (Šimanovci, Belgrade nearby, Serbia). Leaves were placed in plastic Petri dishes (90 mm in diameter) on moist filter paper and incubated at 24 ± 1 °C. After larvae started to hatch, fresh potato leaves were added daily. Four days from hatching larvae entering the 2nd instar were randomly selected from different clusters and uniformly distributed onto potato leaves in different experimental groups.

For insect feeding, fully expanded potato leaves spanning the middle third of field-grown plants were placed on moist filter paper in plastic Petri dishes in a controlled environment room (16 h light: 8 h dark photoperiod, temperature of 24 ± 1 °C, relative humidity of 60 ± 5%). Each leaf was uniformly coated, using a paintbrush, with 200 µL of French marigold flowers MeOH extract in concentrations of 1 or 10 mg of dry crude extract dissolved in 1 mL of deionized water (T1 and T10, respectively). For controls, potato leaves were coated in the same way with 200 µL of deionized water. After complete evaporation of the solvents, five larvae, previously starved for 8 h, were placed on a single potato leaf in a Petri dish, and three Petri dishes were used for each experimental group (*n* = 15). Larvae were fed for the next 9 days until their feeding rate slowed and they entered the prepupal stage. Fresh leaves, coated with either extract or water, were provided daily.

#### 4.5.2. CPB Growth and Development

The larval masses and molting events of individual larvae were measured and monitored daily to determine biomass accumulation and the duration of each developmental stage. After larval development (2nd, 3rd, and 4th instars) had finished, the durations of prepupal and pupal stages were recorded, as well as the coincidence of successfully-finished development (by the number of emerged adults).

#### 4.5.3. CPB Protease Activity Assay

The activity of digestive proteases was analyzed in 3rd and 4th instars reared from the 2nd instar on potato leaves coated with the MeOH extract of French marigold flowers (or with deionized water for controls), as described above.

For extraction of digestive enzymes, the whole larvae were homogenized in 0.9% NaCl (1:5 *w*/*v*) [89] on ice (3 × 10 s at 20,000 rpm) (Ika-Werk Ultra turrax) and sonicated for 3 × 15 s. After that, the homogenate was centrifuged at 5000 ×*g* for 10 min and then the resulting supernatant was centrifuged at 16,000×*g* for 20 min at 4 °C. Bovine serum albumin was used as the standard for the determination of total protein concentration according to Bradford [90].

The method described by Michaud et al. [91] was used for the determination of total protease activity using azocaseine as the substrate at pH 6.5. Crude protein extracts (20 µL) were preincubated with 100 mM L-cysteine and 10 mM EDTA for 30 min at room temperature. After protease activation, the volume of the reaction mixture was adjusted to 100 µL by adding proteolytic buffer pH 6.5 and the mixture was additionally incubated for 10 min at room temperature. As a substrate for proteolysis, 100 µL of 2% azocasein (w/v) was added and the reaction mixtures were incubated in the water bath at 37 °C for 180 min. Proteolysis was terminated by adding 50 μL of 25% trichloroacetic acid (TCA) to the enzyme–substrate mixture. The undigested azocasein was removed by centrifugation at 10,000×*g* for 10 min. The obtained supernatants were mixed with 1 M NaOH in a 1:1 ratio and absorbances were read at 440 nm. The protease activities were expressed in enzyme units (U) per mg of total protein, where one enzyme unit represents the amount of enzyme required for absorbance change of 1.0 during 1 h under the conditions of the assay. Four to six larvae per experimental group were pooled and analyzed as one sample. Each sample was processed in three technical replicates.

### 4.6. Statistical Analyses

Peak picking from the resolved chromatograms was performed using the enviPick R package, while peak correspondence across samples was performed using the density method available in the xcms R package [92]. Moreover, accurate component mass was calculated by using ChemDraw software (version 12.0, CambridgeSoft, Cambridge, MA, USA). Xcalibur software (version 2.1, Thermo Fisher Scientific, Waltham, MA, USA) was used for instrument control, data acquisition, and data analysis.

For the determination of TPC and antioxidant potential (DPPH, ABTS, and FRAP tests), all spectrophotometric measurements were performed in three technical replicates (*n* = 3) for each extract, and values were statistically analyzed using one-way analysis of variance (ANOVA). Differences between the corresponding means were compared using Fisher’s least significant difference (LSD) post hoc test at a significance level of *p* ≤ 0.05. The correlation between TPC in extracts and their antioxidant potential measured by DPPH, ABTS, and FRAP tests was determined using Pearson’s correlation coefficient (R^2^).

For the insect antifeedant bioassay, each experimental group consisted of three biological replicates (Petri dishes) with five CPB larvae within each (*n* = 15). For proteolytic activity measurements, pooled samples of four to six larvae for both 3rd and 4th instars, reared separately from larvae for the abovementioned experiment, were analyzed in technical triplicates (*n* = 3). Means of body mass daily gain and digestive proteolytic activity for each experimental group (insects fed with leaves coated with flower methanol extract in concentrations of 1 mg/mL–T1 and 10 mg/mL–T10) were compared to the control group (insects fed with control leaves coated with deionized water) by Student’s *t*-test for each day of feeding or larval stage, respectively (at *p* ≤ 0.05 or *p* ≤ 0.1). All statistical analyses were performed using Statistica ver. 8.00 software (StatSoft Inc., Tulsa, OK, USA).

## 5. Conclusions

French marigold, as a fast-growing, widespread, easy-to-cultivate species, can serve as a potential high-yielding plant source for the industrial exploitation and production of a “green” alternative for a future insecticide formulation against CPB. The methanol extract of the flowers is rich in phenolics, especially flavonoids. This extract showed antifeedant activity against 3rd and 4th CPB instars, along with strong antioxidant potential. The results suggest methanol extracts of French marigold flowers could be promoted as potent antifeedants, but further testing on non-target animals, along with phytotoxicity and human health issues, should be investigated in more detail.

## Figures and Tables

**Table 1:** The total phenolic content (TPC) and antioxidative activity, measured by 1,1-diphenyl-2-picrylhydrazyl (DPPH), 2,2′-azino-bis(3-ethylbenzothiazoline-6-sulphonic acid (ABTS) and ferric reducing antioxidant power (FRAP) assays, of French marigold flowers and leaves extracts. Correlation between antioxidative potential and TPC was presented as Pearson’s coefficient (R^2^) for each assay.

		TPC (mg GAE/g)	DPPH IC_50_ (mg/mL)	ABTS IC_50_ (mg/mL)	FRAP IC_50_ (mg/mL)
Flowers	Water	37.31 ab	0.404 a	1.023 b	0.120 bc
	Methanol	81.56 c	0.690 a	0.489 a	0.044 ab
	Dichloromethane	37.34 ab	1.722 b	2.158 c	0.231 d
Leaves	Water	39.88 ab	3.176 c	1.694 bc	0.180 c
	Methanol	53.00 b	0.152 a	1.436 bc	0.096 ab
	Dichloromethane	29.84 a	5.465 d	2.578 d	0.299 d
	Trolox	/	0.013 a	0.103 a	0.010 a
R^2^		/	−0.551	−0.799	−0.820

Within each column, values with the same letter are not significantly different at the *p* ≤ 0.05 level according to the LSD test. R^2^ = 0, no correlation; 0 < R^2^ < 1, positive correlation; −1 < R^2^ < 0, negative correlation.

## Data Availability

Data sharing is not applicable to this article.

## Supplementary Materials

The following supporting information can be downloaded at: https://www.mdpi.com/article/10.3390/plants11030407/s1, Figure S1: UHPLC-OrbiTrap MS chromatograms of French marigold flowers and leaves extracts obtained in water, methanol (MeOH), and dichloromethane (DCM); Figure S2: Proposed fragmentation pathway of tagenol B (compound **87**); Table S1: UHPLC-OrbiTrap MS data of phenolics identified in French marigold extracts; Table S2: Peak areas from the full scan MS spectra for identified phenolics in French marigold flowers and leaves extracts obtained in water, methanol (MeOH), and dichloromethane (DCM).
