# Supplementary material for: UHPLC-OrbiTrap MS Characterization of Phenolic Profiles in French Marigold Extracts and Analysis of Their Antifeedant Activity against Colorado Potato Beetle
Source: Plants (Basel). 2022 Feb 1;11(3):407. doi: 10.3390/plants11030407 (PMC8839140; doi:10.3390/plants11030407)
Supplement: Supplementary file 1 [file plants-11-00407-s001.zip › Figure S2.pdf]

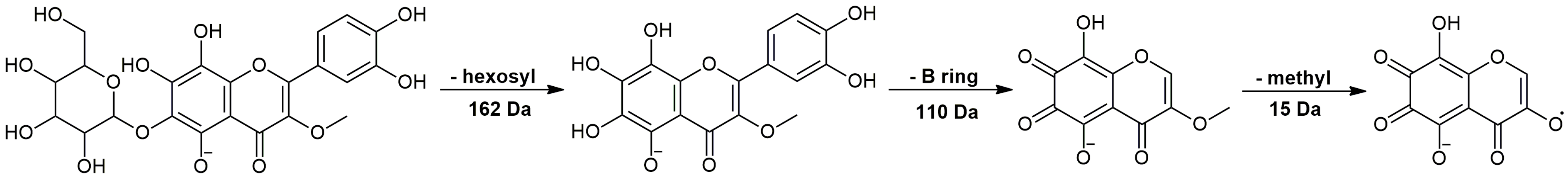

Molecular ion
$C_{22}H_{21}O_{14}^{-}$
509.09368 *m/z*

MS^2 base peak
$C_{16}H_{11}O_{9}^{-}$
347 *m/z*

MS^3 base peak
$C_{10}H_{5}O_{7}^{-}$
237 *m/z*

MS^4 base peak
$C_{9}H_{2}O_{7}^{\bullet-}$
222 *m/z*
