# Supplementary material for: UHPLC-OrbiTrap MS Characterization of Phenolic Profiles in French Marigold Extracts and Analysis of Their Antifeedant Activity against Colorado Potato Beetle
Source: Plants (Basel). 2022 Feb 1;11(3):407. doi: 10.3390/plants11030407 (PMC8839140; doi:10.3390/plants11030407)
Supplement: Supplementary file 1 [file plants-11-00407-s001.zip › Table S1 revised 2.pdf]

Supplementary Table S1. UHPLC-OrbiTrap MS data of phenolics identified in French marigold extracts.

No	Compound name	t_R, min	Molecular formula, $[M-H]^-$	Calculated mass, $[M-H]^-$	Exact mass, $[M-H]^-$	Δ ppm	MS^2 Fragments, (% Base Peak)	MS^3 Fragments, (% Base Peak)	MS^4 Fragments, (% Base Peak)	References (DOI)
	Phenolic acids									
**1**	Galloylquinic acid I	1.58	$C_{14}H_{15}O_{10}^{-}$	343.06707	343.06650	1.68	125(10), 167(3), **169**(100), 170(13), 191(90), 192(8), 297(7)	**125**(100)	81(19), **107**(100), 169(3)	10.3390/molecules26051201
**2**	Gallic acid hexoside	1.94	$C_{13}H_{15}O_{10}^{-}$	331.06707	331.06649	1.76	125(15), **169**(100), 170(9), 271(4)	**125**(100)		10.3390/nu10122002
**3**	Gallic acid	2.34	$C_{7}H_{5}O_{5}^{-}$	169.01425	169.01411	0.84	84(3), 123(6), **125**(100), 126(11)	62(36), 81(47), **97**(100)		10.1002/cbdv.201600463
**4**	Galloylquinic acid II	2.36	$C_{14}H_{15}O_{10}^{-}$	343.06707	343.06649	1.69	169(7), **191**(100), 192(7)	85(80), 93(45), 109(15), 111(44), **127**(100), 171(28), 173(57)	83(19), 85(52), 99(18), **109**(100)	10.3390/molecules26051201
**5**	Dihydroxybenzoic acid hexoside	3.82	$C_{13}H_{15}O_{9}^{-}$	315.07216	315.07165	1.61	108(10), 109(12), 151(8), 152(43), **153**(100), 163(9), 165(12)	**109**(100)		
**6**	Caffeoylglucaric acid I	3.95	$C_{15}H_{15}O_{11}^{-}$	371.06199	371.06149	1.35	191(14), 192(3), **209**(100), 210(8), 315(15), 335(4), 353(15)	85(12), 147(3), **191**(100)	**85**(100), 111(11), 129(6), 145(3), 147(29), 173(21)	10.1016/j.chroma.2016.04.043
**7**	Caffeoylglucaric acid II	4.29	$C_{15}H_{15}O_{11}^{-}$	371.06199	371.06138	1.64	191(18), **209**(100), 210(5), 335(3), 353(11), 354(3)	85(10), 147(3), **191**(100)	**85**(100), 111(5), 129(15), 147(13), 173(45)	10.1016/j.chroma.2016.04.043
**8**	Protocatechuic acid	4.31	$C_{7}H_{5}O_{4}^{-}$	153.01933	153.01928	0.34	107(3), 108(4), **109**(100), 110(8)	**81**(100)		10.1039/C5AY03256C
**9**	Digalloylquinic acid I	4.43	$C_{21}H_{19}O_{14}^{-}$	495.07803	495.07775	0.56	169(22), 325(37), **343**(100), 344(19), 411(6), 447(9), 457(11)	125(19), **169**(100), 173(12), 191(33), 325(4)	**125**(100)	
**10**	3-O-Caffeoylquinic acid	4.58	$C_{16}H_{17}O_{9}^{-}$	353.08781	353.08705	2.14	135(10), 173(4), 179(41), **191**(100), 192(9), 199(23), 318(5)	85(66), 93(35), 109(29), 111(44), **127**(100), 171(38), 173(83)		10.1016/j.jff.2012.05.002
**11**	Caffeic acid hexoside I	4.60	$C_{15}H_{17}O_{9}^{-}$	341.08781	341.08751	0.88	135(56), **179**(100), 180(10), 281(10), 293(25), 295(9), 305(30)	**135**(100), 151(5)		10.1021/np020018i
**12**	Caffeoylglucaric acid III	4.68	$C_{15}H_{15}O_{11}^{-}$	371.06199	371.06158	1.10	191(21), 197(6), 199(11), **209**(100), 210(6), 335(4), 353(9)	85(10), 147(3), **191**(100)	**85**(100), 111(3), 129(3), 147(16), 173(7)	10.1016/j.chroma.2016.04.043
**13**	Digalloyl-dihexoside	4.77	$C_{26}H_{29}O_{19}^{-}$	645.13085	645.12989	1.49	271(15), 313(24), 465(26), 475(42), 483(41), **493**(100), 494(27)	169(28), 211(10), 223(3), 271(74), **313**(100), 314(7), 331(15)	107(6), 123(5), 125(20), **169**(100), 211(10), 251(5), 253(5)	10.3390/molecules26051201
**14**	Digalloylquinic acid II	4.83	$C_{21}H_{19}O_{14}^{-}$	495.07803	495.07797	0.11	169(4), 191(3), 325(9), **343**(100), 344(11), 345(3)	125(13), **169**(100), 191(99)	**125**(100)	
**15**	Hydroxybenzoic acid hexoside	5.00	$C_{13}H_{15}O_{8}^{-}$	299.07724	299.07714	0.33	**137**(100), 138(8)	**93**(100)		
**16**	Syringoyl-galloyl hexoside I	5.03	$C_{22}H_{23}O_{14}^{-}$	511.10933	511.10927	0.11	169(76), 183(22), 271(19), **313**(100), 314(15),	125(9), 151(3), **169**(100), 179(4), 241(11), 253(6),	**125**(100)	10.3390/molecules26051201

							347(12), 373(14)	295(6)		
**17**	Caffeic acid hexoside II	5.15	$C_{15}H_{17}O_9^-$	341.08781	341.08730	1.48	135(8), 162(3), **179**(100), 180(8), 181(9), 295(3)	**135**(100)	**135**(100)	10.1021/np020018i
**18**	Syringoyl-galloyl hexoside II	5.24	$C_{22}H_{23}O_{14}^-$	511.10933	511.10912	0.41	168(12), **169**(100), 183(25), 271(32), 285(10), 313(91), 467(14)	**125**(100)		10.3390/molecules26051201
**19**	5-O-Caffeoylquinic acida	5.25	$C_{16}H_{17}O_9^-$	353.08781	353.08750	0.87	179(3), **191**(100), 192(7), 305(3)	85(86), 93(60), 109(27), 111(33), **127**(100), 171(30), 173(90)	**85**(100), 99(34), 109(39)	10.1016/j.jff.2012.05.002
**20**	Trigalloylquinic acid	5.52	$C_{28}H_{23}O_{18}^-$	647.08899	647.08845	0.84	343(4), 477(12), **495**(100), 496(21), 497(4), 609(13), 610(4)	169(19), 191(3), 193(4), 289(3), 325(46), **343(**100), 477(4)	125(8), **169**(100), 173(11), 191(19)	
**21**	Dicaffeoylaltraric acid I	5.65	$C_{24}H_1O_{14}^-$	533.09368	533.09407	-0.73	209(8), 353(3), **371**(100), 372(13)	191(13), **209**(100), 353(8)	**85**(15), 173(3), 191(100)	10.3390/metabo10100407
**22**	Caffeic acid pentoside I	5.66	$C_{14}H_{15}O_8^-$	311.07724	311.07698	0.82	131(4), 135(7), **149**(100), 150(5), 178(3), 179(93), 180(6)	59(6), 73(5), 88(6), 89(42), 103(18), **131**(100), 134(3)	59(55), 73(37), 87(19), **103**(100)	
**23**	Ellagic acid hexoside	5.73	$C_{20}H_{15}O_{13}^-$	463.05181	463.05209	-0.60	300(22), **301**(100), 302(15)	185(39), 201(14), 229(67), **257**(100), 258(17), 284(38), 301(39)	173(6), **185**(100), 201(10), 213(20), 229(13), 229(78), 438(7)	10.3390/molecules26051201
**24**	Caffeic acid	5.76	$C_9H_7O_4^-$	179.03498	179.03508	-0.56	**135**(100), 136(6)	79(7), 91(13), 93(5), **107**(100), 117(5), 135(44)		10.1002/cbdv.201600463
**25**	Syringoyl-methylgalloyl hexoside I	5.76	$C_{23}H_{25}O_{14}^-$	525.12498	525.12452	0.88	168(6), **183**(100), 184(9), 197(7), 225(7), 285(21), 327(10)	124(9), 139(64), **168**(100)	**124**(100)	10.3390/molecules26051201
**26**	Caffeoyl-(iso)citric acid	5.77	$C_{15}H_{13}O_{10}^-$	353.05142	353.05105	1.06	111(15), 155(7), **173**(100), 174(3), 191(72)	**111**(100), 129(4), 155(29)	**67**(100)	10.1002/jssc.201900407
**27**	Caffeic acid pentoside II	5.91	$C_{14}H_{15}O_8^-$	311.07724	311.07710	0.44	131(3), 135(9), **149**(100), 150(6), 179(74), 180(6)	73(4), 89(39), 103(15), **131**(100)	59(23), 73(25), **103**(100)	
**28**	Syringoyl-methylgalloyl hexoside II	5.94	$C_{23}H_{25}O_{14}^-$	525.12498	525.12539	-0.79	168(11), **183**(100), 184(12), 285(16), 327(8), 479(10), 487(9)	124(9), 139(65), **168**(100)	**124(**100)	10.3390/molecules26051201
**29**	Methyl chlorogenate	6.19	$C_{17}H_{19}O_9^-$	367.10346	367.10337	0.24	173(6), **191**(100), 192(9), 193(5)	85(42), 93(41), 109(36), 111(28), **127**(100), 171(33), 173(38)	**85**(100), 99(87)	10.1016/j.micpath.2017.05.048
**30**	Dicaffeoylaltraric acid II	6.25	$C_{24}H_1O_{14}^-$	533.09368	533.09391	-0.43	191(5), 209(14), 339(5), 353(12), 370(12), **371**(100), 372(13)	191(19), **209**(100), 353(9)	85(18), 147(4), **191**(100)	10.3390/metabo10100407
**31**	Coumaric acid hexoside	6.31	$C_{15}H_{17}O_8^-$	325.09289	325.09276	0.39	101(9), 119(84), 120(11), 161(38), 162(13), **163**(100), 164(10)	**91**(100)		
**32**	Dicaffeoylaltraric acid III	6.40	$C_{24}H_1O_{14}^-$	533.09368	533.09372	-0.07	191(4), 209(13), 353(10), 369(3), **371**(100), 372(15), 487(3)	191(16), **209**(100), 353(13)	85(19), 147(5), 173(3), 191(15), **191**(100)	10.3390/metabo10100407
**33**	Disyringoyl hexoside I	6.40	$C_{24}H_{27}O_{14}^-$	539.14063	539.14086	-0.42	182(12), 183(15), **197**(100), 198(10), 341(86), 342(14), 479(8)	121(6), 138(5), **153**(100), 182(70)	121(27), **138**(100)	10.3390/molecules26051201

**34**	Mehylellagic acid hexoside	6.51	$C_{21}H_{17}O_{13}^-$	477.06746	477.06781	-0.73	300(6), **315**(100), 316(13), 317(3)	**300**(100), 301(5)	200(48), 228(41), 243(38), 244(75), 271(41), 272(31), **300**(100)	
**35**	Disyringoyl hexoside II	6.56	$C_{24}H_{27}O_{14}^-$	539.14063	539.14080	-0.31	182(14), 183(11), **197**(100), 198(11), 341(82), 342(14), 479(7)	121(5), 138(5), 153(71), **182**(100)	123(4), 138(13), **167**(100)	10.3390/molecules26051201
**36**	Coumaroyl-(iso)citric acid	6.62	$C_{15}H_{13}O_9^-$	337.05651	337.05645	0.16	111(11), 155(4), **173**(100), 191(6)	**111**(100), 129(3), 155(26)	**67**(100), 183(4)	
**37**	Feruloyl-(iso)citric acid	6.70	$C_{16}H_{15}O_{10}^-$	367.06707	367.06730	-0.64	111(9), 155(5), **173**(100), 174(3)	**111**(100), 129(3), 155(28)	**67**(100)	10.1002/jssc.201900407
**38**	Ellagic acida	6.74	$C_{14}H_5O_8^-$	300.99899	300.99909	-0.32	185(26), 229(36), 255(20), **257**(100), 271(54), 284(27), 301(43)	157(18), **185**(100), 201(27), 213(25), 229(92), 242(17), 257(17)		10.3390/molecules26051201
**39**	Vanillic acid	6.83	$C_8H_7O_4^-$	167.03498	167.03515	-1.02	97(10), 108(38), 121(43), 122(13), 123(13), 130(13), **152**(100)	83(4), **108**(100), 124(3)	73(31), **94**(100), 124(36)	10.3390/molecules22020313
**40**	Dicaffeoylquinic acid I	7.11	$C_{25}H_{23}O_{12}^-$	515.11950	515.11938	0.23	**353**(100), 354(14), 447(5), 477(6)	135(9), 173(4), 179(40), **191**(100)	85(79), 93(61), 109(14), 111(38), **127**(100), 171(30), 173(57)	10.3390/molecules26051201
**41**	Dicaffeoylquinic acid II	7.26	$C_{25}H_{23}O_{12}^-$	515.11950	515.11905	0.88	203(9), 299(6), 315(5), **353**(100), 354(16), 477(37), 478(10)	135(12), **173**(100), 179(83), 191(93)	57(8), 59(15), **93**(100), 109(11), 111(84), 137(8), 155(19)	10.3390/molecules26051201
**42**	Tricaffeoylglucaric acid	7.45	$C_{33}H_{27}O_{17}^-$	695.12537	695.12593	-0.81	209(4), 353(5), 371(42), 372(6), 515(7), **533**(100), 534(23)	191(6), 209(18), 353(11), 371(21), **371**(100)	191(16), **209**(100), 353(10)	10.3390/metabo11040220
**43**	Methylellagic acid	7.82	$C_{15}H_7O_8^-$	315.01464	315.01444	0.65	235(3), **300**(100), 301(16)	200(44), 216(22), 228(27), 243(23), 244(93), 271(61), **300(**100)		
**44**	Ferulic acid	7.99	$C_{10}H_9O_4^-$	193.05063	193.05057	0.30	129(4), 134(5), **147**(100), 148(5), 149(9), 150(3), 178(5)	75(9), 99(5), 101(7), 115(11), 117(6), 119(9), **129**(100)	55(10), 57(35), **85**(100), 123(16)	10.3390/molecules22020313
**45**	Dimethylellagic acid hexoside	8.71	$C_{22}H_{19}O_{13}^-$	491.08311	491.08313	-0.03	**329**(100), 330(23), 331(19), 342(18), 371(9), 464(5), 476(4)	283(7), 286(32), 291(4), 301(20), 311(5), **314(**100), 315(7)	242(3), 258(19), **286**(100)	
**46**	Dimethylellagic acid	10.50	$C_{16}H_9O_8^-$	329.03029	329.02968	1.85	286(19), 287(3), 301(8), **314(**100), 315(9), 316(5)	258(7), **286**(100)	202(19), 230(27), 242(5), **258**(100), 269(3), 286(4)	
	Flavonoids									
**47**	Quercetagetin	7.35	$C_{15}H_9O_8^-$	317.03029	317.03013	0.50	167(58), 195(49), 245(39), **271**(100), 287(45), 299(79), 300(51)	183(4), 199(68), 215(11), 227(18), 241(3), **243**(100)	171(11), 187(13), 198(5), **199**(100), 201(4), 215(68), 225(4)	10.1007/s13197-016-2228-6
**48**	Luteolin[a]	8.65	$C_{15}H_9O_6^-$	285.04046	285.04048	-0.06	175(69), 199(67), 217(55), 241(80), 243(45), **285**(100), 286(32)	**197**(100), 198(91), 199(83), 212(10), 213(52), 214(15), 226(24)	142(3), 153(35), 155(3), **169**(100), 182(34), 198(11), 288(8)	10.3390/molecules22020313
**49**	6-Hydroxykaempferol	8.73	$C_{15}H_9O_7^-$	301.03538	301.03502	1.17	177(18), 255(23), 257(38), 271(41), 273(20), **283**(100), 284(19)	201(3), 211(6), 227(9), 239(27), **255**(100), 265(3), 283(3)	182(4), 183(8), 187(23), 209(5), 211(93), **227**(100), 255(12)	10.1021/acs.jafc.0c02042

**50**	8-Hydroxy-3-O-methylquercetagetin	8.78	$C_{16}H_{11}O_{19}$	347.04086	347.04041	1.28	193(32), 209(35), 237(51), 286(18), 329(34), 330(28), **332**(100)	166(35), 195(35), **286**(100), 287(61), 288(49), 303(76), 304(21)	202(8), 214(5), 230(11), 241(7), 257(16), **258**(100), 259(10)	
**51**	Patuletin	8.78	$C_{16}H_{11}O_{8}^{-}$	331.04594	331.04539	1.65	166(3), 181(3), 314(3), **316**(100), 317(10)	166(90), 243(36), 255(25), 270(57), 271(85), **287**(100), 288(42)	215(40), 231(25), 241(22), 243(47), 258(19), **259**(100), 287(14)	10.1007/s11094-021-02387-z
**52**	Apigenin[a]	9.52	$C_{15}H_{9}O_{5}^{-}$	269.04555	269.04547	0.28	149(55), 151(73), 181(19), 201(37), **225**(100), 227(24), 269(67)	157(7), 169(15), 180(12), **181**(100), 183(44), 196(22), 197(47)		
**53**	Kaempferol[a]	9.68	$C_{15}H_{9}O_{6}^{-}$	285.04046	285.04026	0.72	169(15), 185(22), 213(13), 229(18), 239(17), 255(75), **285**(100)	159(50), **183**(100), 184(7), 211(47), 213(20), 227(38), 255(17)		10.3390/molecules26051201
**54**	Chrysoeriol[a]	9,74	$C_{16}H_{11}O_{6}^{-}$	299.05611	299.05614	-0.11	217(10), **284**(100), 285(17)	**256**(100), 257(3), 284(11), 285(3)	158(19), 188(16), 199(16), 211(21), 212(18), **227**(100), 228(19)	http://www.orientjchem.org/vol12no3/chrysocriol-7-06-o-%CE%B1-l-arabinofuranosyl-%CE%B2-d-glucopyranoside-from-tagetes-patula/
**55**	6-Methoxykaempferol	9,79	$C_{16}H_{11}O_{7}^{-}$	315.05103	315.05078	0.78	272(5), 300(6), **300**(100), 301(11)	166(6), 254(4), 255(10), 256(6), 271(31), **272**(100)	199(4), 225(4), 227(5), 243(13), 254(50), **255**(100), 271(11)	https://www.researchgate.net/publication/276272147_Ivancheva_St_Zdravkova_M_1993_Flavonoids_in_Tagetes_patula
**56**	Axillarin	10,06	$C_{17}H_{13}O_{8}^{-}$	345.06159	345.06120	1.13	287(3), 302(10), 329(5), **330**(100), 331(18), 332(4)	287(49), 301(9), **302**(100), 312(12), 315(17)	284(3), 285(5), 286(34), **287**(100)	https://www.researchgate.net/publication/281677527_Flavonoids_from_the_flowers_of_Tagetes_erecta_L
**57**	5,7-Dimethylquercetin	10,17	$C_{17}H_{13}O_{7}^{-}$	329.06668	329.06608	1.82	171(4), **314**(100), 315(16), 316(9)	271(6), **299**(100), 300(3)	165(3), 166(3), 227(9), 243(7), 255(12), **271**(100)	http://en.cnki.com.cn/Article_en/CJFDTOTAL-HXYO200704003.htm
**58**	8-Hydroxyquercetagetin	11,11	$C_{15}H_{9}O_{9}^{-}$	333.02521	333.02469	1.55	**315**(100), 316(21), 317(6), 318(14)	**300**(100), 301(3)	216(8), 244(23), **272**(100)	10.3390/molecules26051201
	Flavonoid glycosides									
**59**	Quercetagetin 3,7-di-*O*-hexoside	4.88	$C_{27}H_{29}O_{18}^{-}$	641.13594	641.13557	0.57	317(39), 318(8), 477(4), 478(24), **479**(100), 480(37), 481(6)	315(4), 316(39), **317**(100)	167(47), 195(79), 208(40), 231(42), 245(44), **271**(100), 299(89)	10.1039/C5AY02186C
**60**	Quercetagetin 3-*O*-pentoside-7-*O*-hexoside	4.93	$C_{26}H_{27}O_{17}^{-}$	611.12537	611.12476	1.00	316(10), 317(23), **449**(100), 450(19), 478(18), 479(21), 563(4)	309(3), **316**(100), 317(87), 329(3)	139(26), **166**(100), 194(32), 255(34), 270(50), 271(92), 287(59)	10.1016/S0378-8741(97)00038-X
**61**	Quercetin 3,7-di-*O*-hexoside	5.13	$C_{27}H_{29}O_{17}^{-}$	625.14102	625.14083	0.32	301(22), 462(6), 462(19), **463**(100), 464(24), 465(5), 563(5)	299(8), 300(55), **301**(100), 343(6)	117(9), **151**(100), 179(79), 193(38), 255(36), 257(20), 273(38)	10.17660/ActaHortic.1999.501.34
**62**	Patuletin 3,7-di-*O*-hexoside I	5.20	$C_{28}H_{31}O_{18}^{-}$	655.15159	655.15160	-0.01	331(14), 373(3), 492(17), **493**(100), 494(24), 495(7), 535(4)	315(16), 316(8), 329(5), 330(41), **331**(100), 373(10), 478(10)	271(4), 287(4), 288(3), **316**(100)	10.1007/s11094-021-02387-z
**63**	Quercetin 3-*O*-pentoside-7-*O*-hexoside	5.22	$C_{26}H_{27}O_{16}^{-}$	595.13046	595.13007	0.66	301(27), **433**(100), 434(27), 462(80), 463(65), 464(13), 475(9)	151(3), 179(3), 271(3), **300**(100), 301(35), 343(7)	179(3), 227(3), 243(3), 254(13), 255(12), **271**(100), 272(12)	

**64**	Patuletin 3-*O*-pentoside-7-*O*-hexoside	5.32	$C_{27}H_{29}O_{17}^{-}$	625.14102	625.14097	0.08	331(10), **463**(100), 464(16), 492(80), 493(78), 494(14), 505(6)	300(9), 301(10), 315(36), **330**(100), 331(40), 373(6), 448(22)	180(9), 287(5), 301(5), 312(26), **315**(100), 316(20)	
**65**	6-Methoxykaempferol 3-*O*-pentoside-7-*O*-hexoside	5.49	$C_{27}H_{29}O_{16}^{-}$	609.14611	609.14586	0.41	**447**(100), 448(24), 449(4), 463(4), 476(16), 477(14), 489(17)	285(4), 299(44), 300(6), **314**(100), 315(23), 357(19), 432(60)	179(5), 180(5), 268(6), 271(11), 285(3), 286(3), **299**(100)	
**66**	Patuletin 3,7-di-*O*-hexoside II	5.86	$C_{28}H_{31}O_{18}^{-}$	655.15159	655.15179	-0.32	331(5), 479(5), **493**(100), 494(28), 607(4), 609(44)	316(4), 330(6), **331**(100), 373(4)	288(3), **316**(100)	10.1007/s11094-021-02387-z
**67**	Quercetin 3-*O*-hexoside-7-*O*-rhamnoside	5.90	$C_{27}H_{29}O_{16}^{-}$	609.14611	609.14576	0.57	301(26), 431(7), 446(22), **447**(100), 448(30), 463(58), 464(18)	299(3), 300(5), **301**(100)	107(8), **151**(100), 179(63), 229(12), 255(19), 273(11), 301(11)	
**68**	Patuletin 3-*O*-(2''-hexosyl)hexoside	5.99	$C_{28}H_{31}O_{18}^{-}$	655.15159	655.15168	-0.13	315(26), 316(32), **330**(100), 331(80), 332(21), 475(29), 493(42)	287(6), **315**(100), 316(37)	175(10), 227(5), 243(8), 245(10), 259(9), 271(20), **287**(100)	10.1007/s11094-021-02387-z
**69**	Quercetin 3-*O*-rhamnoside-7-*O*-pentoside	6.06	$C_{26}H_{27}O_{15}^{-}$	579.13554	579.13561	-0.11	301(26), 433(99), **446**(100), 447(73)	**299**(100)		
**70**	Quercetin 3-*O*-(6''-hexosyl)hexoside	6.07	$C_{27}H_{29}O_{17}^{-}$	625.14102	625.14123	-0.33	271(6), 300(37), **301**(100), 302(12), 343(10), 463(4), 579(45)	151(64), **179**(100), 229(6), 256(7), 257(14), 272(8), 273(15)	**151**(100)	10.17660/ActaHortic.1999.501.34
**71**	Quercetagetin 6-*O*-hexoside	6.13	$C_{21}H_{19}O_{13}^{-}$	479.08311	479.08347	-0.74	315(6), 316(7), **317**(100), 318(22)	139(47), 167(68), 195(39), 227(33), 243(35), **271**(100), 299(44)	145(5), 187(11), 199(72), 215(22), 227(20), 242(5), **243**(100)	10.1016/j.phytol.2016.04.004
**72**	Quercetin 3-*O*-(6''-galloyl)hexoside-7-*O*-hexoside	6.14	$C_{34}H_{33}O_{21}^{-}$	777.15198	777.15349	-1.94	**615**(100), 616(25)	301(13), **463**(100)	300(3), 300(46), **301**(100), 343(3)	10.1515/znc-2014-4165
**73**	Patuletin 3-*O*-(6''-hexosyl)hexoside	6.20	$C_{28}H_{31}O_{18}^{-}$	655.15159	655.15200	-0.62	287(12), 316(19), 330(11), **331**(100), 332(17), 373(6), 493(16)	287(3), **316**(100), 317(3)	166(7), 243(4), 244(7), 270(61), 271(46), **287**(100), 288(49)	10.1007/s11094-021-02387-z
**74**	Quercetin 3-*O*-(6''-pentosyl)hexoside	6.23	$C_{26}H_{27}O_{16}^{-}$	595.13046	595.13121	-1.26	300(43), **301**(100), 302(15), 343(11), 447(70), 448(18), 463(8)	107(6), 151(85), **179**(100), 256(16), 257(22), 272(27), 273(25)	**151**(100)	10.1111/php.12619
**75**	Quercetin 3,7-di-*O*-rhamnoside	6.30	$C_{27}H_{29}O_{15}^{-}$	593.15119	593.15146	-0.46	301(27), 302(4), 446(28), **447**(100), 448(22), 449(4), 555(4)	300(25), **301**(100), 343(3)	107(20), **151**(100), 179(56), 229(37), 255(56), 273(26), 283(20)	
**76**	Kaempferol 3-*O*-pentoside-7-*O*-rhamnoside	6.32	$C_{26}H_{27}O_{14}^{-}$	563.14063	563.14038	0.44	285(12), **417**(100), 418(22), 419(4), 430(58), 431(32), 432(6)	151(3), 255(8), **284**(100), 285(19), 327(14)	227(14), **255**(100), 256(22)	
**77**	Kaempferol 3-*O*-hexoside-7-*O*-pentoside	6.35	$C_{26}H_{27}O_{15}^{-}$	579.13554	579.13544	0.19	255(16), 284(47), **285**(100), 286(15), 327(16), 447(63), 448(13)	151(23), 229(40), 239(21), 241(37), 256(57), **257**(100), 267(41)	161(29), 163(44), 185(22), 213(26), **229**(100), 239(32), 240(15)	
**78**	Kaempferol 3-*O*-(6''-hexosyl)hexoside	6.37	$C_{27}H_{29}O_{16}^{-}$	609.14611	609.14585	0.43	257(9), 284(10), 284(27), **285**(100), 286(14), 327(16), 463(16)	151(36), 213(26), 229(46), 241(37), 256(32), **257**(100), 267(52)	163(67), 173(5), 185(10), 189(16), 213(38), **229**(100), 239(23)	10.1016/0305-1978(87)90079-2
**79**	Quercetin 3-*O*-(6''-galloyl)hexoside	6.40	$C_{28}H_{23}O_{16}^{-}$	615.09916	615.09872	0.71	300(3), 301(15), 451(3), **463**(100), 464(21), 579(86), 580(3)	300(38), **301**(100)	151(76), **179**(100), 255(9), 257(12), 271(8), 273(15), 283(5)	10.1016/0031-9422(92)80489-2

**80**	6-Methoxykaempferol 3-*O*-(6''-hexosyl)hexoside	6.51	$C_{28}H_{31}O_{17}^{-}$	639.15667	639.15624	0.67	255(8), 271(21), 272(12), 300(30), **315**(100), 316(22), 331(21)	**300**(100)	166(4), 254(6), 255(14), 256(3), 271(49), **272**(100)	
**81**	Patuletin 7-*O*-(6''-galloyl)hexoside	6.51	$C_{29}H_{25}O_{17}^{-}$	645.10972	645.11088	-1.79	330(5), 331(16), 483(5), **493**(100), 494(26), 495(5), 609(4)	316(5), 330(23), **331**(100)	181(3), **316**(100)	
**82**	Kaempferol 3-*O*-rhamnoside-7-*O*-rhamnoside	6.65	$C_{27}H_{29}O_{14}^{-}$	577.15628	577.15577	0.88	285(33), 286(5), **431**(100), 432(19), 433(3), 531(11)	284(12), **285**(100)	213(88), 215(61), 229(66), 241(75), **243**(100), 257(78), 285(82)	10.1016/j.biopha.2017.06.064
**83**	Quercetin 3-*O*-glucoside[a]	6.67	$C_{21}H_{19}O_{12}^{-}$	463.08820	463.08854	-0.74	299(3), 300(22), **301**(100), 302(9)	151(77), **179**(100), 255(17), 257(12), 271(28), 272(15), 273(18)	**151**(100)	10.1002/cbdv.201700415
**84**	Kaempferol 3-*O*-rhamnoside-7-*O*-pentoside	6.73	$C_{26}H_{27}O_{14}^{-}$	563.14063	563.14165	-1.80	285(6), 417(14), **431**(100), 432(22), 493(13)	**285**(100)	213(51), 239(38), 241(38), 243(77), **257**(100), 267(49), 285(61)	
**85**	Kaempferol 7-*O*-(6''-galloyl)hexoside	6.74	$C_{28}H_{23}O_{15}^{-}$	599.10424	599.10439	-0.24	285(17), 313(33), 447(36), 551(7), 553(10), **563**(100), 564(20)	285(11), 417(7), **431**(100)	**285**(100)	
**86**	Patuletin 7-*O*-hexoside	6.75	$C_{22}H_{21}O_{13}^{-}$	493.09877	493.09900	-0.47	**331**(100), 332(13), 373(4)	287(3), **316**(100), 317(3)	244(6), 270(60), 271(41), **287**(100), 288(40)	
**87**	8-Hydroxy-3-O-methyl quercetagetin 6-O-hexoside	6.76	$C_{22}H_{21}O_{14}^{-}$	509.09368	509.09417	-0.96	345(13), 346(14), **347**(100), 371(14), 451(18), 463(10), 491(14)	191(9), 193(63), 209(9), **237**(100), 238(9), 329(20), 332(55)	166(3), 194(6), 209(16), **222**(100)	
**88**	Quercetin 3-*O*-galloyl-rhamnoside-7-*O*-rhamnoside	6.95	$C_{34}H_{33}O_{19}^{-}$	745.16215	745.16282	-0.90	301(41), 302(7), 446(7), **447**(100), 448(22), 599(16), 600(4)	**301**(100)	107(13), **151**(100), 179(70), 229(6), 255(10), 273(11), 301(13)	
**89**	Kaempferol 3-*O*-glucoside[a]	6.97	$C_{21}H_{19}O_{11}^{-}$	447.09916	447.09650	5.94	231(24), 255(16), **284**(100), 285(68), 316(22), 317(17), 327(15)	227(13), **255**(100), 256(16)	167(4), **211**(100), 213(4), 227(78), 255(22)	10.1016/0305-1978(94)90034-5
**90**	Quercetin 3-*O*-pentoside	7.00	$C_{20}H_{17}O_{11}^{-}$	433.07764	433.07798	-0.79	299(5), 300(24), **301**(100), 302(19)	151(80), **179**(100), 255(7), 257(10), 272(8), 273(20), 283(15)	**151**(100)	10.3390/molecules24213911
**91**	Patuletin 3-*O*-pentoside	7.05	$C_{21}H_{19}O_{12}^{-}$	463.08820	463.08846	-0.55	300(8), 301(23), 315(5), 329(11), 330(94), **331**(100), 332(18)	181(3), 315(6), **316**(100), 317(3)	166(12), 243(11), 270(49), 271(46), 272(14), **287**(100), 288(70)	10.1177/1934578X20974507
**92**	Quercetin 3-*O*-rhamnoside[a]	7.18	$C_{21}H_{19}O_{11}^{-}$	447.09329	447.09417	-1.98	299(6), 300(33), **301**(100), 302(15)	151(84), **179**(100), 229(11), 256(9), 272(9), 273(21), 283(20)	**151**(100)	10.1016/0305-1978(94)90034-5
**93**	Axillarin 7-*O*-(6''rhamnosyl)hexoside	7.25	$C_{29}H_{33}O_{17}^{-}$	653.17232	653.17196	0.56	315(8), 329(19), 330(18), 344(48), **345**(100), 346(18), 607(32)	180(4), 195(14), 223(4), 315(4), **330**(100)	284(3), 285(4), 301(4), 302(5), 312(20), 314(3), **315**(100)	
**94**	Quercetin 3-*O*-(2''-galloyl)-pentoside	7.27	$C_{27}H_{21}O_{15}^{-}$	585.08859	585.08868	-0.15	300(4), **301**(100), 302(14), 433(3)	151(77), **179**(100), 193(7), 229(4), 239(5), 257(12), 273(18)	**151**(100)	10.1371/journal.pone.0198739
**95**	6-Methoxykaempferol 7-*O*-hexoside	7.30	$C_{22}H_{21}O_{12}^{-}$	477.10385	477.10417	-0.67	300(8), 313(4), 314(20), **315**(100), 316(18), 357(9), 462(3)	272(7), **300**(100), 301(4)	166(3), 254(6), 255(14), 256(6), 271(38), **272**(100)	

**96**	Kaempferol 3-*O*-galloyl-rhamnoside-7-*O*-rhamnoside	7.33	$C_{34}H_{33}O_{18}^-$	729.16724	729.16815	-1.25	285(23), 297(22), 430(7), **431**(100), 432(19), 583(19), 584(6)	151(3), 284(23), **285**(100)	**151**(100), 213(31), 229(7), 241(37), 243(11), 257(58), 285(13)	
**97**	Kaempferol 3-*O*-pentoside	7.33	$C_{20}H_{17}O_{10}^-$	417.08272	417.08313	-0.98	179(5), **284**(100), 285(39), 327(15), 361(8), 371(31), 383(11)	227(9), **255**(100), 256(24)	67(3), 167(6), 183(24), 211(96), 213(7), **227**(100), 255(48)	10.3390/molecules24213911
**98**	Luteolin 7-*O*-glucoside[a]	7.42	$C_{21}H_{19}O_{11}^-$	447.09329	447.09401	-1.63	**285**(100), 286(15)	151(36), 175(73), 199(85), 217(67), **241**(100), 243(77), 285(41)	183(17), 197(90), **198**(100), 199(30), 213(63), 214(15), 241(31)	10.1016/0305-1978(94)90034-5
**99**	7-Methylquercetagetin 6-O-hexoside	7.52	$C_{22}H_{21}O_{13}^-$	493.09877	493.09918	-0.84	316(3), 329(17), **331**(100), 332(14), 333(3), 373(3), 447(11)	209(4), **316**(100)	166(78), 243(42), 244(32), 270(33), 271(72), **287**(100), 288(41)	10.1002/star.201500068
**100**	5,7-Dimethylquercetin 3-*O*-rutinoside	7.56	$C_{29}H_{33}O_{16}^-$	637.17741	637.17770	-0.45	271(22), 313(31), 314(15), 328(46), **329**(100), 330(22), 371(19)	195(13), 286(9), 299(6), 301(3), 314(23), **314**(100), 315(5)	180(3), 271(22), 286(73), 287(3), 296(11), **299**(100)	
**101**	Axillarin 7-*O*-hexoside	7.61	$C_{23}H_{23}O_{13}^-$	507.11442	507.11475	-0.67	331(22), 337(13), 344(13), **345**(100), 346(19), 347(10), 492(35)	166(3), **330**(100)	179(4), 243(4), 271(5), 287(81), 312(69), **315**(100), 482(4)	10.1016/j.phytol.2016.04.004
**102**	Kaempferol 3-*O*-(2''-galloyl)-pentoside	7.68	$C_{27}H_{21}O_{14}^-$	569.09368	569.09381	-0.23	283(27), 284(7), **285**(100), 286(13), 435(7), 507(6), 521(9)	**151**(100), 213(19), 229(13), 239(11), 241(41), 257(82), 285(26)	63(4), **107**(100)	10.1038/s41598-019-54546-8
**103**	3,3'-Dimethylquercetin 7-*O*-hexoside	7.72	$C_{23}H_{23}O_{12}^-$	491.11950	491.11965	-0.31	193(11), 314(14), 315(18), **329**(100), 330(19), 476(59), 477(13)	**314**(100), 315(3)	180(3), 270(3), 271(61), 285(3), 286(3), **299**(100)	
**104**	Trimethylquercetagetin rutinoside	7.72	$C_{30}H_{35}O_{17}^-$	667.18797	667.18805	-0.12	329(46), 344(14), **359**(100), 360(19), 493(13), 629(39), 630(16)	316(8), 329(16), **344**(100)	301(30), 316(54), **329**(100)	
**105**	Patuletin 7-*O*-(6''-hydroxybenzoyl)hexoside	7.77	$C_{29}H_{25}O_{15}^-$	613.11989	613.12028	-0.63	316(15), **331**(100), 463(61), 464(15), 491(93), 492(16), 598(23)	181(8), 209(4), 272(7), 288(4), 303(4), **316**(100), 317(6)	188(10), 216(8), 244(9), 270(65), 271(29), **287**(100), 288(54)	
**106**	Axillarin 7-*O*-(6''-caffeoyl)hexoside	8.04	$C_{32}H_{29}O_{16}^-$	669.14611	669.14648	-0.55	287(21), 316(17), 323(36), 330(25), **331**(100), 332(16), 345(49)	181(3), 287(4), **316**(100), 317(7)	166(9), 179(3), 243(7), 270(58), 271(51), **287**(100), 288(52)	
**107**	Axillarin 7-*O*-pentoside	8.05	$C_{22}H_{21}O_{12}^-$	477.10385	477.10417	-0.67	343(3), **344**(100), 345(96), 346(14), 347(3), 387(4), 433(3)	301(7), **329**(100), 330(5)	175(5), 270(5), 285(3), 286(36), 301(26), 311(3), **314**(100)	
**108**	Patuletin 3-*O*-(6''-*p*-coumaroyl)hexoside	8.10	$C_{31}H_{27}O_{15}^-$	639.13554	639.13487	1.05	287(8), 316(20), 331(9), **331**(100), 332(17), 489(6), 517(7)	209(5), 270(3), 287(3), **316**(100), 317(14)	166(9), 243(6), 244(11), 270(54), 271(54), **287**(100), 288(67)	10.1016/S0305-1978(02)00250-8
**109**	Quercetin 3-*O*-(6''-vaniloyl)hexoside	8.26	$C_{29}H_{25}O_{15}^-$	613.11989	613.11902	1.42	299(10), 300(30), **301**(100), 302(15), 315(45), 316(6), 415(13)	151(77), **179**(100), 180(5), 256(6), 257(11), 272(6), 273(25)	**151**(100)	
**110**	Quercetin 3-*O*-(2''-hydroxybenzoyl)-pentoside	8.35	$C_{27}H_{21}O_{13}^-$	553.09877	553.09880	-0.06	299(10), 300(51), **301**(100), 302(26), 303(10), 433(10), 507(16)	121(8), 151(52), 174(6), **179**(100), 228(6), 271(6), 273(12)	150(6), **151**(100), 169(3)	
**111**	Kaempferol 7-*O*-rhamnoside	8.45	$C_{21}H_{19}O_{10}^-$	431.09837	431.09874	-0.86	151(4), 257(3), 283(4), 284(30), **285**(100), 286(16), 393(5)	107(3), **151**(100), 213(16), 241(29), 257(47)	65(3), 83(8), **107**(100)	10.3390/molecules24213911

**112**	Patuletin 7-*O*-(6"-benzoyl)hexoside	8.96	$C_{29}H_{25}O_{14}^{-}$	597.12498	597.12494	0.07	316(18), 331(25), **331**(100), 332(18), 373(12), 479(8), 551(7)	270(3), 287(3), **316**(100), 317(9)	270(56), 271(31), **287**(100), 288(37)	
**113**	Quercetin 3-*O*-(6"-*p*-coumaroyl)hexoside	8.98	$C_{30}H_{25}O_{14}^{-}$	609.12498	609.12469	0.48	299(11), 300(33), **301**(100), 302(18), 315(32), 433(32), 447(11)	151(48), **179**(100), 180(10), 192(7), 229(6), 256(8), 272(24)	**151**(100), 299(4)	10.1002/jms.3982

[a]Confirmed using available standards, all the other compounds were identified based on MS/MS data. [b]Peaks that were further fragmentated in MS^3 and MS^4 experiment are marked bold in table.
