# Supplementary material for: UHPLC-OrbiTrap MS Characterization of Phenolic Profiles in French Marigold Extracts and Analysis of Their Antifeedant Activity against Colorado Potato Beetle
Source: Plants (Basel). 2022 Feb 1;11(3):407. doi: 10.3390/plants11030407 (PMC8839140; doi:10.3390/plants11030407)
Supplement: Supplementary file 1 [file plants-11-00407-s001.zip › Table S2 revised 2.pdf]

Supplementary Table S2. Peak areas from the full scan MS spectra for identified phenolics in French marigold flowers and leaves extracts obtained in water, methanol (MeOH) and dichloromethane (DCM)

		Flowers			Leaves		
		**Water**	**MeOH**	**DCM**	**Water**	**MeOH**	**DCM**
	Phenolic acids / glycosides						
**1**	Galloylquinic acid I	2726763	5684281			1175310	
**2**	Gallic acid hexoside	353607			560503	3408919	
**3**	Gallic acid	17467976	3288340				
**4**	Galloylquinic acid II	7079504	12032618			3901552	
**5**	Dihydroxybenzoic acid hexoside	1639681	832143		21024399	18034291	
**6**	Caffeoylglucaric acid I	1874435					
**7**	Caffeoylglucaric acid II	2605216			223388		
**8**	Protocatechuic acid	30444008	7791086		4458420	2104843	
**9**	Digalloylquinic acid I	189880	219886			2680894	
**10**	3-O-Caffeoylquinic acid	3094608	625839		168874	224667	
**11**	Caffeic acid hexoside I				153964	551547	
**12**	Caffeoylglucaric acid III	2516540			219346		
**13**	Digalloyl-dihexoside	59036	5397848			411834	
**14**	Digalloylquinic acid II	2683052	6870984			4134116	
**15**	Hydroxybenzoic acid hexoside	489754	494786		1408275	3028240	
**16**	Syringoyl-galloyl hexoside I	977505	2437475			489190	
**17**	Caffeic acid hexoside II	382107	524731		342720	1602738	
**18**	Syringoyl-galloyl hexoside II	1464624	5096652			893096	
**19**	5-O-Caffeoylquinic acida	5500260	36174680		1756839	16229651	
**20**	Trigalloylquinic acid		160692			7401045	
**21**	Dicaffeoylaltraric acid I	15697750					
**22**	Caffeic acid pentoside I				317579	1844521	
**23**	Ellagic acid hexoside		4967412			6598921	
**24**	Caffeic acid	43433276	1184675		608233	736138	

**25**	Syringoyl-methylgalloyl hexoside I	719315	1811086				
**26**	Caffeoyl-(iso)citric acid	905022	274949		25380759	16610842	92375
**27**	Caffeic acid pentoside II	595121	995968		2025565	17973326	125634
**28**	Syringoyl-methylgalloyl hexoside II	1608728	4334909		101729	137111	
**29**	Methyl chlorogenate	77044	880566		1756964	2190273	
**30**	Dicaffeoylaltraric acid II	4713779					
**31**	Coumaric acid hexoside	1320969	527512		2523052	756057	
**32**	Dicaffeoylaltraric acid III	4236650	144286				
**33**	Disyringoyl hexoside I	1540743	3031824				71269
**34**	Mehylellagic acid hexoside		2251357			7815412	
**35**	Disyringoyl hexoside II	2463099	6296924	177199	299008	340213	153758
**36**	Coumaroyl-(iso)citric acid	1629759	299576		43954373	16062630	
**37**	Feruloyl-(iso)citric acid	12525886	4508555		127066846	57148007	513137
**38**	Ellagic acida	1207313	11022261			1162901	
**39**	Vanillic acid	3133472	2571448		426515	488697	
**40**	Dicaffeoylquinic acid I	5720797	24025408	128982	102074	15703724	70072
**41**	Dicaffeoylquinic acid II			70239		3747503	
**42**	Tricaffeoylglucaric acid	11619014	7249802			158462	
**43**	Methylellagic acid	340985	3476514		142747	1194839	
**44**	Ferulic acid	257441	417898		4130087	2617146	
**45**	Dimethylellagic acid hexoside		1466942				
**46**	Dimethylellagic acid	2077834	97761721	2402785		1720916	84739
	Flavonoids						
**47**	Quercetagetin	132480	29188850				
**48**	Luteolin[a]	3421116	81625041	185492		1379732	
**49**	6-Hydroxykaempferol	1224978	8529246	809880	315421	3034643	333329
**50**	8-Hydroxy-3-O-methylquercetagetin	1274384	28729475	313606		1930408	
**51**	Patuletin	40618617	337392962	15611804	528291	52811159	1363322
**52**	Apigenin[a]	144153	3604935			271657	

**53**	Kaempferol[a]	417270	2287967	657480	164870	1500441	261625
**54**	Chrysoeriol[a]		2785543				
**55**	6-Methoxykaempferol	360761	37796625	899009		2305050	237161
**56**	Axillarin		2353522			766458	242819
**57**	5,7-Dimethylquercetin					850982	174681
**58**	8-Hydroxyquercetagetin	3323127	22644123				
	Flavonoid glycosides						
**59**	Quercetagetin 3,7-di-*O*-hexoside		502365			2289123	
**60**	Quercetagetin 3-*O*-pentoside-7-*O*-hexoside		167120			830331	
**61**	Quercetin 3,7-di-*O*-hexoside		1796508			4631457	
**62**	Patuletin 3,7-di-*O*-hexoside I		10166420			15362042	
**63**	Quercetin 3-*O*-pentoside-7-*O*-hexoside		694092			6839927	
**64**	Patuletin 3-*O*-pentoside-7-*O*-hexoside		1725930			9301886	
**65**	6-Methoxykaempferol 3-*O*-pentoside-7-*O*-hexoside					1885344	
**66**	Patuletin 3,7-di-*O*-hexoside II		3315580			123926	
**67**	Quercetin 3-*O*-hexoside-7-*O*-rhamnoside		21524649		425620	62476127	510893
**68**	Patuletin 3-*O*-(2''-hexosyl)hexoside	1358159	3011399				
**69**	Quercetin 3-*O*-rhamnoside-7-*O*-pentoside		9442613	61183	1704243	49634014	484744
**70**	Quercetin 3-*O*-(6''-hexosyl)hexoside	988151	8136907		5510269	19696048	119978
**71**	Quercetagetin 6-*O*-hexoside		58972571	147565		12179482	
**72**	Quercetin 3-*O*-(6''-galloyl)hexoside-7-*O*-hexoside		141399			4275669	
**73**	Patuletin 3-*O*-(6''-hexosyl)hexoside	1437344	18091487	206580	222044	3716661	
**74**	Quercetin 3-*O*-(6''-pentosyl)hexoside	1791935	3578742		177888	3345275	
**75**	Quercetin 3,7-di-*O*-rhamnoside		12307193	94847	6708679	54885163	527701
**76**	Kaempferol 3-*O*-pentoside-7-*O*-rhamnoside		255392		235238	9606619	78430
**77**	Kaempferol 3-*O*-hexoside-7-*O*-pentoside	76628	743223		2079529	19682381	205495
**78**	Kaempferol 3-*O*-(6''-hexosyl)hexoside		681257		1435699	20946406	77847

**79**	Quercetin 3-*O*-(6''-galloyl)hexoside	141746	1931768			26401271	103294
**80**	6-Methoxykaempferol 3-*O*-(6''-hexosyl)hexoside	225856	2175059		618228	2853561	
**81**	Patuletin 7-*O*-(6''-galloyl)hexoside	398307	2221821				
**82**	Kaempferol 3-*O*-rhamnoside-7-*O*-rhamnoside		174155		391095	11661628	149661
**83**	Quercetin 3-*O*-glucoside[a]	1001399	11129137	81054	2002753	83837241	766011
**84**	Kaempferol 3-*O*-rhamnoside-7-*O*-pentoside				191295	5875294	
**85**	Kaempferol 7-*O*-(6''-galloyl)hexoside					3697732	
**86**	Patuletin 7-*O*-hexoside	1543862	167730168	2325685		28055167	142103
**87**	8-Hydroxy-3-O-methyl quercetagetin 6-O-hexoside		8104156			583970	
**88**	Quercetin 3-*O*-galloyl-rhamnoside-7-*O*-rhamnoside		220522			5351948	
**89**	Kaempferol 3-*O*-glucoside[a]	638089			1118234	4377422	
**90**	Quercetin 3-*O*-pentoside	2438489	7239489		5748534	46975440	174289
**91**	Patuletin 3-*O*-pentoside	432903	2028365		939071	4467177	
**92**	Quercetin 3-*O*-rhamnoside[a]	1125253	7040595		5617038	7953994	
**93**	Axillarin 7-*O*-(6''rhamnosyl)hexoside				2547040	5696052	
**94**	Quercetin 3-*O*-(2''-galloyl)-pentoside	166544	2281634			25874199	107385
**95**	6-Methoxykaempferol 7-*O*-hexoside		17382518			2768290	
**96**	Kaempferol 3-*O*-galloyl-rhamnoside-7-*O*-rhamnoside					5514139	
**97**	Kaempferol 3-*O*-pentoside				562237	3032392	
**98**	Luteolin 7-*O*-glucoside[a]		10770488		178110	1113854	
**99**	Quercetagetin-7-methyl ether 6-*O*-hexoside	164016	22374573			556642	
**100**	5,7-Dimethylquercetin 3-*O*-rutinoside				1011260	3088918	
**101**	Axillarin 7-*O*-hexoside				100535	5006036	
**102**	Kaempferol 3-*O*-(2''-galloyl)-pentoside					2853731	
**103**	3,3'-Dimethylquercetin 7-*O*-hexoside		94477			3167728	

**104**	Trimethylquercetagetin rutinoside				643353	1867861
**105**	Patuletin 7-*O*-(6''-hydroxybenzoyl)hexoside		2006097			
**106**	Axillarin 7-*O*-(6''-caffeoyl)hexoside	65828	2701602			
**107**	Axillarin 7-*O*-pentoside					1079968
**108**	Patuletin 3-*O*-(6''-*p*-coumaroyl)hexoside		1403542			102815
**109**	Quercetin 3-*O*-(6''-vaniloyl)hexoside				101985	1412093
**110**	Quercetin 3-*O*-(2''-hydroxybenzoyl)-pentoside		231934			898591
**111**	Kaempferol 7-*O*-rhamnoside				136930	659536
**112**	Patuletin 7-*O*-(6''-benzoyl)hexoside		1173062			
**113**	Quercetin 3-*O*-(6''-*p*-coumaroyl)hexoside		226625			598889
