# Supplementary figures and images for: UHPLC-OrbiTrap MS Characterization of Phenolic Profiles in French Marigold Extracts and Analysis of Their Antifeedant Activity against Colorado Potato Beetle
Source: Plants (Basel). 2022 Feb 1;11(3):407. doi: 10.3390/plants11030407 (PMC8839140; doi:10.3390/plants11030407)

RT: 0.00 - 20.01 SM: 5G

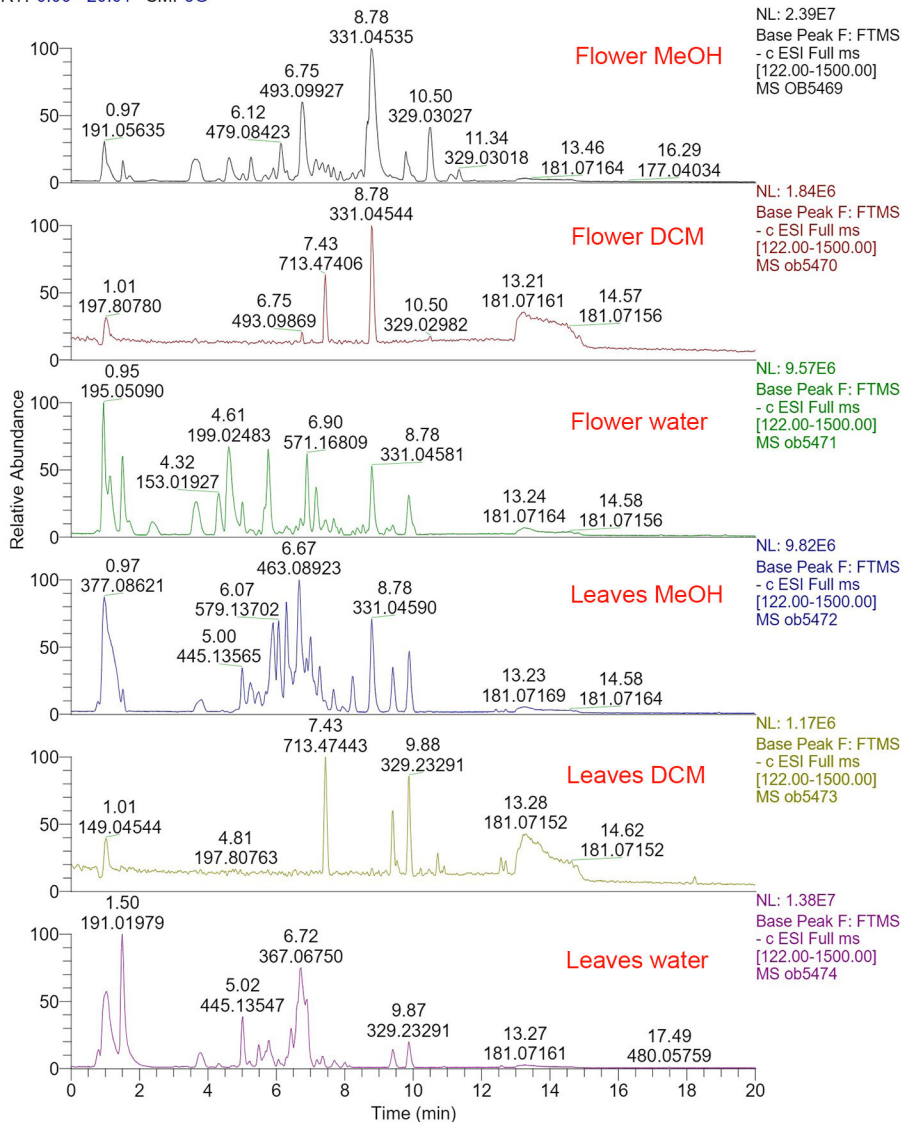

Supplement: Supplementary file 1 [file plants-11-00407-s001.zip › Figure S1.pdf]
